# The Neuroprotective Potential of *Ocimum* Plant Species: Seasoning the Mind with Sweet and Holy Basil

**DOI:** 10.3390/nu17172877

**Published:** 2025-09-05

**Authors:** Alexandru Vasincu, Răzvan-Nicolae Rusu, Daniela-Carmen Ababei, Delia Bulea, Oana Dana Arcan, Ioana Mirela Vasincu, Sorin Beșchea Chiriac, Ionuț-Răducu Popescu, Walther Bild, Veronica Bild

**Affiliations:** 1 Department of Pharmacodynamics and Clinical Pharmacy, “Grigore T Popa” University of Medicine and Pharmacy, 16 Universitatii Street, 700115 Iasi, Romania; alexandru.vasincu@umfiasi.ro (A.V.); dana.ababei@gmail.com (D.-C.A.); delia.bulea@umfiasi.ro (D.B.); oana-dana.arcan@umfiasi.ro (O.D.A.); veronica.bild@gmail.com (V.B.); 2 Department of Pharmaceutical Chemistry, “Grigore T Popa” University of Medicine and Pharmacy, 16 Universitatii Street, 700115 Iasi, Romania; ioana-mirela.vasincu@umfiasi.ro; 3 Department of Veterinary Toxicology, “Ion Ionescu de la Brad” University of Life Sciences, 8 M. Sadoveanu Alley, 700489 Iasi, Romania; sbeschea@yahoo.com; 4 Department of Physiology, “Grigore T Popa” University of Medicine and Pharmacy, 16 Universitatii Street, 700115 Iasi, Romania; ionutraducu@gmail.com (I.-R.P.); waltherbild@gmail.com (W.B.); 5 “Olga Necrasov” Center of Anthropological and Biomedical Research, Romanian Academy, Iasi Branch, 700506 Iasi, Romania

**Keywords:** neurodegenerative disease, Alzheimer’s disease, *Ocimum* sp., oxidative stress, lipid peroxidation

## Abstract

Neurodegenerative diseases (NDs) encompass a group of chronic conditions, characterized by neuronal losses in large areas of the brain, leading to cognitive and behavioral impairments. Alzheimer’s Disease (AD), the most common form of dementia, is a progressive ND, characterized by the accumulation of amyloid β and tau protein, entails cognitive decline, neuroinflammation, mitochondrial dysfunction, and blood–brain barrier impairment, with oxidative stress playing a critical role in its pathogenesis. To date, the available pharmacotherapy has shown limited efficacy, and multitarget activity of plant-derived neuroprotective bioactive compounds is currently in focus. This review synthesizes experimental evidence regarding *Ocimum* species with neuroprotective potential in AD, particularly *Ocimum sanctum* and *Ocimum basilicum*. These plants are rich in bioactive compounds including polyphenols, flavonoids, essential oils, and triterpenoids that synergistically scavenge reactive oxygen/nitrogen species, upregulate endogenous antioxidant enzymes (SOD, CAT, and GPx), and reduce lipid peroxidation. Furthermore, these extracts have demonstrated the ability to decrease β-amyloid accumulation and tau protein levels, key pathological features of AD. Even though additional research is required to fully assess their potential as therapeutic agents for NDs, by diving into the specific mechanisms through which they improve neurodegenerative processes, important steps can be made towards this endpoint.

## 1. Introduction

Neurodegenerative diseases (NDs) are a group of chronic conditions that involve multiple dysfunctions at motor, sensory, and perceptual levels that finally lead to cognitive and behavioral impairments. These diseases are progressive and characterized by neuronal loss in large areas of the brain, mostly seen among the elderly [1,2].

Oxidative stress, glycation, aberrant protein aggregation, inflammatory processes, and gradual neuronal death are some common features shared by NDs, including Alzheimer’s disease (AD), Parkinson’s disease (PD), Huntington’s disease (HD), or Amyotrophic lateral sclerosis (ALS). The brain is especially susceptible to oxidative stress because of its high rate of oxygen consumption and weak detoxifying systems [3]. Age-related changes in cellular activity provide predisposition pathways for a number of illnesses, including AD, making aging the main risk factor for NDs.

The present review aims to explore the neuroprotective effects of *Ocimum* sp., especially *Ocimum sanctum* (*O. sanctum*) and *Ocimum basilicum* (*O. basilicum*), in AD. In addition, it focuses on the involvement of oxidative stress in neurodegeneration and the role of antioxidants identified in these plants in the inhibition of this pathophysiological process. The brain is considered the most vulnerable organ to oxidative stress due to high oxygen consumption and low regenerative capacity. By examining the mechanisms through which these compounds exert their neuroprotective effects, this review seeks to provide insights in the designing of possible therapeutic applications for the treatment of neurodegenerative diseases.

## 2. Review Methodology

A comprehensive literature search was conducted across multiple databases, including PubMed, Scopus, Web of Science, and ScienceDirect, using keywords such as “*Ocimum*”, “*Tulsi*”, “*Holy basil*”, “*O. sanctum*”, “*O. basilicum*”, “phytochemicals”, “neuroprotection”, “Alzheimer’s disease”, and “oxidative stress”. Inclusion criteria comprised studies published in English between 2000 and 2025 that focused on phytochemical analysis as well as in vitro, in vivo, or clinical evidence highlighting the neuroprotective properties of *Ocimum* species. Exclusion criteria encompassed studies that did not specifically address neurodegeneration or were limited to agronomic or chemotaxonomic investigations lacking references to biomedical data. This enhances transparency and reduces the risk of selection bias.

## 3. Mechanisms of Neurodegeneration

The current literature proposes several possible mechanisms of the neurodegenerative process. The mitochondria have been identified as a major source of reactive oxygen species (ROS) production, and its dysfunction over time appears to contribute to neuronal degradation and the aging process [4]. A key feature of this theory is its potential to influence both the pace and the underlying mechanisms of aging [5]. Mitochondria represents the primary site of cellular oxygen metabolism, predominantly originating from Complex I, via partial reduction in the FMN (flavin mononucleotide) moiety bound to nicotinamide adenine dinucleotide reduced (NADH) dehydrogenase, and from Complex III, where the semi-reduced forms of ubiquinone/ubisemiquinone/ubiquinol participate in electron transfer reactions with molecular oxygen [6]. In line with this concept, inhibitors of mitochondrial respiratory complexes are routinely used to trigger oxidative stress in both in vitro and in vivo experimental models of NDs, providing valuable tools for pharmacological investigations.

Another relevant mitochondrial contributor to ROS production is the enzyme family of monoamine oxidases (MAOs), anchored to the outer mitochondrial membrane. MAOs catalyze the oxidation of biogenic amine neurotransmitters—including norepinephrine, dopamine, and serotonin (5-hydroxytryptamine)—a process that inherently generates free radicals. MAO-B, in particular, mediates the oxidation of 1-methyl-4-phenyl-1,2,3,6-tetrahydropyridine (MPTP) to its toxic metabolite MPP^+^, which exerts Complex I inhibition and elicits biochemical, clinical, and neuropathological alterations resembling idiopathic PD [7].

Mitochondria are not the exclusive source of ROS relevant to neurodegeneration. Transition metals can stimulate free radical formation, and oxidative stress markers appear prior to pathological lesions in AD [8]. In AD brains, elevated copper levels together with decreased iron have been linked to activation of multiple oxidative pathways, including protein kinase C (PKC, EC 2.7.11.13), NADPH oxidases (EC 1.6.3.1), extracellular signal-regulated kinase (ERK) 1/2 (EC 2.7.11.24), and poly (ADP-ribose) polymerase (PARP, EC 2.4.2.30), promoting oxidative neuronal necrosis [9].

## 4. Role of Oxidative Stress in Neurodegenerative Diseases

Oxidative stress plays a pivotal role in the pathogenesis of multiple NDs. Emerging evidence suggests intricate interconnections between oxidative stress, neuroinflammation, mitochondrial dysfunction, and other neurodegenerative processes, both at disease onset and during progression [10].

The process of oxidation involves electron loss, a process that must be precisely regulated within living systems to preserve redox equilibrium. However, unstable molecules such as ROS, continuously generated during cellular metabolism, can propagate chain reactions by destabilizing adjacent molecules. Overproduction of ROS contributes to aging and degenerative pathologies, prompting the evolution of endogenous antioxidant defense systems, including superoxide dismutase (SOD), catalase (CAT), reduced glutathione (GSH), and glutathione peroxidase (GPx). In compromised antioxidant systems, ROS accumulation induces oxidative stress, triggering pro-apoptotic Bcl-2 family protein activation and thereby initiating the programmed cell death pathway. Bcl-2 proteins regulate apoptosis by either inhibiting (anti-apoptotic) or promoting (pro-apoptotic) mitochondrial cytochrome c release [11].

The human brain is particularly susceptible to oxidative damage due to its high polyunsaturated fatty acid (PUFA) content, including arachidonic and linoleic acids. Lipid peroxidation, initiated by ROS-PUFA interactions, generates lipid peroxyl radicals that propagate further oxidative chain reactions. Antioxidant systems mitigate this by scavenging free radicals and interrupting peroxidation cascades [12]. Key ROS implicated in neurodegeneration include superoxide anion (O_2_^−^), hydrogen peroxide (H_2_O_2_), and hydroxyl radical (HO•). Reactive nitrogen species (RNS), such as nitric oxide (NO), also contribute by reacting with O_2_^−^ to form peroxynitrite (ONOO^−^), a potent oxidant that decomposes into HO• [13].

The widely accepted oxidative stress theory of aging posits that age-related functional decline stems from cumulative oxidative damage to lipids, DNA, and proteins by ROS/RNS [7]. Elevated ROS/RNS levels may induce cellular senescence, characterized by pro-inflammatory factor secretion and extracellular matrix degradation. Chronic oxidative stress disrupts homeostatic regulation, particularly in immune and antioxidant systems, while simultaneously activating inflammatory pathways. This creates a self-perpetuating cycle where inflammation exacerbates oxidative stress, further accelerating neurodegeneration [14].

Under physiological conditions, nitric oxide (NO) enhances mitochondrial biogenesis via cyclic GMP (cGMP)-dependent activation of peroxisome proliferator-activated receptor gamma coactivator-1α (PGC-1α). However, excessive nitrosative stress impairs mitochondrial respiration through S-nitrosylation of complexes I and IV in the electron transport chain [15].

Oxidative stress is a hallmark of early neurodegenerative stages, with ROS overproduction correlating with cognitive decline and neuroinflammation. Preclinical studies suggest that targeted antioxidant interventions or ROS-scavenging strategies may reduce inflammatory markers and mitigate neuronal damage [16,17]. Breaking the oxidative stress–inflammation cycle represents a promising therapeutic avenue for halting disease progression.

## 5. Pathophysiology of Alzheimer’s Disease

AD is the most common disorder of the central nervous system (CNS), characterized by the destruction of neurons and synaptic transmissions, with progressive and irreversible evolution. Key neuropathological hallmarks include the accumulation of β-amyloid (Aβ) plaques and neurofibrillary tangles in the brain. These changes lead to progressive deterioration of memory, cognition, and behavioral function, which represent the core clinical manifestations of AD [18,19,20,21,22,23].

The hippocampus, a component of the limbic system, plays a central role in memory process, especially episodic and autobiographical memory. In AD, hippocampal neurogenesis is disrupted due to altered neuropeptide Y signaling, which is essential for synaptic plasticity and neuronal survival [24].

The Aβ peptide is generated through proteolytic cleavage of the amyloid precursor protein (APP), that occurs under the action of β- and γ-secretase, yielding Aβ42 (42-aminoacid peptide) and Aβ40 peptide, which is shorter by 2 amino acids at the C-terminal end. The aggregation of Aβ fragments leads to plaque formation, which subsequently contributes to the cholinergic neurotransmission impairment observed in the brains of AD patients [20]. Imbalances between Aβ production and clearance mechanisms lead to its accumulation, facilitating plaque formation and synaptic toxicity [18,21].

Aβ aggregates disrupt cholinergic neurotransmission and interact with copper (Cu^+^) and zinc (Zn^2+^) in the synaptic cleft, promoting oxidative crosslinking and amyloid precipitation [25]. While Zn^2+^ plays a physiological role in modulating intracellular signaling pathways, particularly those governing protein phosphorylation [26] as well as oxidative stress defenses [27], dysregulated Zn^2+^ homeostasis can also precipitate mitochondrial impairment and activate apoptotic pathways, thereby exacerbating neuronal loss in AD [28].

Oxidative stress, neuroinflammation, tau protein phosphorylation, various neurotransmitters such as cholinergic, and abnormal energy metabolism have been observed to play an important role in the development of neuropathology of AD, characterized by impaired cognitive function, especially memory impairment, which is an irreversible process [29,30,31].

A very important aspect is the blood–brain barrier (BBB), as pathological changes in the blood–brain barrier lead to disruption of physiological functions with induction of brain damage and contribute to many neurodegenerative diseases such as AD [32,33]. Accumulation of Aβ has been associated with elevated prostaglandin levels leading to inflammatory processes and blood–brain barrier dysfunction [34].

It is known that the incidence and prevalence of severe cognitive impairment are increasing, as global life expectancies continue to rise. Around the world, more than 55 million people are estimated to be living with AD or other dementias as of recent years, with this number expected to rise to over 152 million by 2050 [35]. AD currently affects around 7.2 million Americans aged 65 and older and this number is expected to increase to 13.8 million by 2060 without substantial medical advancements in prevention or treatment [36]. Poor quality treatment leads to increased pressure on healthcare infrastructures, imposing significant socioeconomic burdens across affected populations [37]. Developing new methods, including multitarget-directed ligands (MTDLs) that can simultaneously target numerous pathogenic pathways that contribute to neurodegeneration and can delay the onset and progression of NDs, is a paramount objective with huge socioeconomic value [38].

## 6. Botanical and Ethnopharmacological Profile of *Ocimum* sp.

Natural compounds were the first agents used in a therapeutic purpose. Numerous researchers suggest they possess neuroprotective effects, highlighting their potential for managing disorders such as AD [39]. The plant species *Ocimum*, collectively called basil, is considered to be the largest genera in this family and most of the species are native to temperate regions of the globe [40]. The plant is highly distributed in Africa, South America (Brazil) and Asia (India) [41]. For example, in India, nine species of *Ocimum* have been reported and three of these are exotic: *O. americanum* L., *O. minimum* L., and *O. africanum* Lour. [42]. Various authors reported between approximately 30 and 160 species of annual and perennial herbs and shrubs [43]. However, The Plant List database includes 384 scientific plant names of species rank for the genus *Ocimum* and only 76 of these are accepted species names, the rest being considered as synonyms, unplaced or unassessed [44]. This could be explained due to the high degree of polymorphism of the plants [45]. Extensive cultivation and different environmental factors have led to morphological variations seen in *Ocimum* species [46].

The most frequently cultivated *Ocimum* species as a source of essential oils and aromatic compounds [47] in some countries of Europe, America, East Asia, and Australia are *Ocimum basilicum* (*O. basilicum* or sweet basil), *Ocimum gratissimum* (*O. gratissimum*), and *Ocimum sanctum (O. sanctum* or holy basil) [48]. *Ocimum americanum* (*O. americanum*) is met as ubiquitous species in India but also is cultivated in Indonesia due to its essential oil. *Ocimum kilimandscharicum* (*O. kilimandscharicum*) is cultivated due to its camphor-like scent essential oil [41].

## 7. Phytochemicals from *Ocimum* sp. with Anti-AD Potential

The phytochemical analysis of basil showed that this plant is rich in polyphenolic compounds and flavonoids which play an important role in free radical scavenging. The total polyphenol and flavonoid contents can vary among different *Ocimum* species, with the highest concentrations found in *O. basilicum* and *O. gratissimum*, while the lowest concentrations were found in *O. canum*. The high level of the active compounds was correlated with important antioxidant activity, as measured by various radical scavenging assays [49,50]. Usually, polyphenol content was measured in mg gallic acid equivalent per gram of extract (mg GAE/g), while flavonoid content was measured in mg quercetin equivalent per gram of extract (mg QE/g) [49,51].

The predominant polyphenolic compounds identified in *Ocimum* extracts include sinapic acid, rosmarinic acid, methyl eugenol, eugenol, luteolin, apigenin, ocimarin, nepetoidin, xanthomicrol, hymenoxin, luteolin-7-O-glucuronide, chlorogenic acid, salvigenin, apigenin-7-O-glucuronide, basilimoside, and oleanolic acid [52]. These compounds highlighted potent radical scavenger activity. Furthermore, there is proof that indicates anti-inflammatory activity in *Ocimum* extracts, demonstrated by the inhibition of lipoxygenase enzyme and nitric oxide radical scavenging, enzymes known to be critical in regulating inflammatory responses in various disease conditions [49,53].

Mass spectrometry coupled with liquid or gas chromatography is the most widely used method for identifying natural compounds. For *Ocimum* species, various extraction methods using different solvents such as water–ethanol, ethanol, methanol, ethyl acetate, water, alcohol–acid, chloroform, and hexane, were used [50,52].

*O. sanctum* contains several phytochemicals, including flavonoids, terpenoids, phenolic compounds, glycosides, tannins, saponins, alkaloids, and steroids. Additionally, it is rich in vitamins C and A, and minerals such as zinc, iron, and calcium, but also in fatty acids or carboxylic acids, aliphatic aldehyde, and amino acid or carbohydrate derivatives [50,54].

*O. sanctum* exhibits notable inhibitory effects on lipid peroxidation that incriminate the higher concentration of polyphenolic flavonoids and phenolics, which are strong antioxidants. Studies have shown that ethanolic extract of *O. sanctum* significantly strengthens antioxidant defense mechanisms [55,56], demonstrating its neuroprotective properties against noise-induced oxidative stress. In animal models, this is demonstrated by elevated GSH, CAT, and SOD activity [57,58].

According to experimental studies, ethanolic extracts of *O. sanctum* could improve memory function in mouse models, by modifying the cholinergic pathways in the brain. Both assays used, scopolamine-induced and aging-induced amnesia, could exhibit higher step-down latency and suppression of acetylcholinesterase activity in human brain microvascular endothelial cells indicating a mechanism based on acetylcholine control [59,60]. The ability of the extract to combat oxidative stress through bioactive components such as ursolic acid, rosmarinic acid, flavonoids (like luteolin and apigenin), and polyphenolic compounds (like cirsimaritin and cirsilineol) further supports its neuroprotective effects [57,58,61].

It seems that the moieties that contain phenolic -OH groups could be correlated with antioxidant capacity. By stabilizing phenoxy radicals, electron-donating substituents (like alkyl and hydroxyl) increase antioxidant activity, whereas electron-withdrawing groups (like COOH) decrease efficacy. If we are referring to flavonoids, it is known that catechol structure promotes electron donation, which strengthens radical scavenging and also improve membrane protection [55,62].

Regarding *O. basilicum*, its leaf oil contains important components such as methyl chavicol (estragole), linalool, methyl cinnamate, 1,8-cineole, and methyl eugenol. Ethanolic or methanolic extract may include flavonoids (luteolin, catechin, vitexin apigenin, naringenin, and rutin), rosmarinic acid, phenols, anthocyanins, and steroids [63]. It seems that apigenin and C-glycosylated derivatives could have some potential activity in AD as anti-inflammatory molecules. Also, naringenin has exhibited some antioxidant activity [64]. Studies suggest that *O. basilicum* can mediate GABAergic neurotransmission and reduce brain acetylcholinesterase level and oxidative stress, thereby improving cognitive functions [65].

Studies that involved various species of *Ocimum* (*O. minimum*, *O. americanum*, *O. africanum*, and *O. basilicum*) which aimed to prove their anticholinesterase activity were developed. The preliminary results reported that *O. americanum* showed the highest activity, then *O. africanum* followed by *O. basilicum* and *O. minimum*. The major components identified were phenolics; rosmarinic, caftaric, and chlorogenic acids of which chlorogenic acid demonstrated the highest activity [66].

The extracts of *O. basilicum* were made using various solvents such as water, ethyl acetate, methanol, ethanol, butanol, chloroform, and diethyl-ether. These demonstrated notable inhibition of lipid peroxidation (LPx) in liposomal models and exhibited strong free radical scavenging activity. The essential oils of *O. basilicum* displayed greater antioxidant potential than its individual constituents, suggesting a synergistic interaction among its components that enhances hydrogen-donating capacity. The main components of the essential oil are eugenol and methyl chavicol. It seems that the introduction of methoxy groups could increase the antioxidant capacity of eugenol. Not only the phenolic components are the most important but also the metabolites that showed an antioxidant effect similar to reference [67].

Table 1 and Table 2 summarize the phytochemicals reported by several groups of researchers in different *Ocimum* species that possess neuroprotective effects as well as their chemical structures.

## 8. Neuroprotective Mechanisms of *Ocimum* sp. Phytochemicals

Neuronal cells are particularly vulnerable to oxidative stress due to the brain’s elevated oxygen consumption, which underscores the critical importance of antioxidants in preventing or delaying neurodegeneration [169].

Recent studies have identified various compounds in spices, including *O. sanctum* and *O. basilicum*, that exhibit positive modulatory properties in synaptic transmissions [20]. Dietary intake rich in antioxidants has been associated with a decreased risk of dementia, with *O. sanctum* recognized for its antioxidant and other therapeutic properties, such as antidiabetic, antifungal, antimicrobial, antineoplastic, cardioprotective, analgesic, and diaphoretic effects [30]. *O. basilicum* is traditionally used for various nervous disorders, but also neurodegenerative diseases. A large number of studies reveal the potential of basil to modulate important neurotransmissions such as the GABAergic but especially the cholinergic one by reducing brain acetylcholinesterase (AChE) activity [65,170]. The efficacy of *O. americanum* L. extracts has been evaluated by their inhibitory action on cholinesterases (AChE and butyrylcholinesterase) as well as free radical scavenging and reduction [126].

A major component of AD pathophysiology is oxidative stress, which leads to DNA damage, lipid peroxidation, and progressive cell death. Natural antioxidants can inhibit free radical formation through numerous mechanisms and reduce amyloid plaque accumulation [171,172]. The antioxidant action of basil extract has been found to restore SOD and metalloproteinases, chelate Fe^2+^, and ameliorate hydrogen peroxide-induced neuronal damage [173,174].

The antioxidant activity of *O. sanctum* has been widely associated with neuroprotection and has been documented by numerous researchers [175]. The antioxidant and neuroprotective activity of *O. sanctum* compared to that of the natural product EGb761, was evaluated in a rat model of cerebral ischemia induced by bilateral common carotid artery occlusion (BCCAO), when motor dysfunction was attenuated, and the size of cerebral infarction was reduced. ROS uptake may be due to phenolic compounds, tannins, and flavonoids present in the *O. sanctum* extract [176].

In vivo studies on Wistar albino rats indicate that *O. sanctum* protects against cadmium-induced toxicity by enhancing the activity of endogenous antioxidant enzymes such as SOD, CAT, GPx, GSH, and ascorbic acid (vitamin C) [177]. These effects have been attributed to the antioxidant properties of flavonoids, which play an essential role in membrane protection. Compounds like orientin and vicenin from *O. sanctum* have demonstrated significant in vivo antioxidant effects, reducing γ-radiation-induced lipid peroxidation in mouse liver. A significant decrease in the MDA levels beginning from 15 min to 8 h post-irradiation compared to control mice was observed [178]. Lipid peroxidation, driven by oxidative stress, is a critical pathway leading to neuronal membrane damage. Other phenolic compounds, including cirsilineol, cirsimaritin, apigenin, and rosmarinic acid also contribute to the antioxidant and neuroprotective properties of *O. sanctum* extract of fresh leaves and stems [179]. Epigenol, isotimonin, and isothymusine showed antioxidant activity as compared to butylated hydroxy toluene (BHT) and tert-butyl hydroquinone (TBHQ) taken as standards [180]. These bioactive compounds not only neutralize free radicals but may also modulate multiple pathways involved in oxidative damage and neuroinflammation.

The essential oils extracted from *O. sanctum* leaves are particularly rich in eugenol, carvacrol, thymol, linalool, caryophyllene, limatrol, apigenin, and ursolic acid; among these, eugenol is considered the most therapeutically valuable [181]. These compounds show promise as therapeutic candidates for NDs. The antioxidant properties of basil essential oils containing these compounds were compared with other known antioxidants used as standards, such as BHT and tocopherol, showing notable activity [174].

The question of how eugenol through its antioxidant action reduces the inflammatory response arises. This plant-derived bioactive compound has been shown to prevent amyloid plaque formation in the hippocampus as well as amyloid-induced hemolysis [182,183]. It also inhibited microsomal lipid peroxidation in rat liver mitochondria [55,184]. In fact, neutralization of reactive oxygen and nitrogen species, and inhibition of lipid peroxidation that prevails the antioxidant effect, are mechanisms of action which underlie the functional and biological activity of eugenol [185].

As previously discussed, neuronal cells are particularly susceptible to oxidative damage [169]. When antioxidant defense systems become impaired, the resulting oxidative stress can contribute significantly to neurodegenerative conditions. Moreover, oxidative stress and inflammation are mutually reinforcing phenomena. In inflamed tissues, increased ROS levels are frequently observed, creating a vicious cycle that exacerbates tissue damage [186], emphasizing the importance of antioxidants in both the prevention and therapeutic management of neurological diseases.

Notably, *O. sanctum* extract has been shown to regulate neurotransmitter levels critical for neuronal function. In a rat model of AD, the extract both alone and in combination with levetiracetam improved cognitive function, reduced motor dysfunction, and decreased the size of cerebral infarctions. The extract also significantly reduced tau protein and beta-amyloid levels in the hippocampus, restoring histological and neurochemical changes induced by Aβ [187].

Cognitive dysfunction induced in animal models (rats) by the administration of substances such as atropine or cyclosporine or by maximal electroshock, was improved by the administration of *O. sanctum* extract, decreasing the brain AChE activity [188]. In another study, an ethanolic extract of *O. sanctum* improved cognitive ability of rats by acting on the cholinergic system, increasing the activity of choline acetyltransferase (ChAT) activity, which is responsible for the synthesis of acetylcholine (ACh). Twenty-seven white male rats in different ages, 3, 6, and 9 months, were treated with an ethanolic extract of *O. sanctum* for 45 days in different doses. The evaluation of the behavioral study by the 8-arm radial maze, in the assessment of long-term memory, showed an improvement in cognitive ability in 6- and 9-month-old rats treated with 100 mg/kg b.w. of the extract. Moreover, in 6-month-old rats, the extract increased the expression of ChAT responsible for the synthesis of ACh in the brain [60].

Chronic brain hypoperfusion can be caused by free radicals through the generation of brain damage, leading to histopathological and functional disorders. The active principles of *O. sanctum* such as eugenol, isotimucine, rosmarinic acid, orientin, vicenin, and apigenin, have been shown to be able to function as potent antioxidant and neuroprotective pharmacophores. Tested on an animal model (rats) of cerebral ischemia and hypoperfusion, *O. sanctum* extract (200 mg/kg b.w./day) used for 7 days has prevented increased lipid peroxidation and oxidative stress [176].

Another preclinical study in animal models (rats) demonstrated that *O. sanctum* ethanolic extract had a neuroprotective effect by increasing the density of hippocampal neurons. The AD model was induced using the chemical agent trimethyltin (TMT), which causes lesions mainly in the hippocampal area, causing the rats to show certain behavioral symptoms. The administration of *O. sanctum* extract suggests an increase in neuropeptide expression and prevents neuronal apoptosis [24].

The neuroprotective effect of *O. sanctum* extract has been demonstrated by increasing the viability of human embryonic kidney-293 (HEK-293) cells and maintaining the stability of ChAT expression. For induction of the neurodegeneration and neurotoxicity model, cell cultures were treated with TMT and pre-treatment with *O. sanctum* extract provided protection against apoptosis [189].

Not only *O. sanctum* demonstrated its neuroprotective effect, but also the extract from the *O. gratissimum* species leaves that were administered in Wistar rats with cerebral ischemia. The extract improved cognitive performance, especially the parameters that define anxiety [69]. The antioxidant activity of *O. gratissimum* was attributed to flavonoids and phenolic compounds that prevent the induction of oxidative stress generated by cell damage. Blocking of enzyme systems and scavenging free radicals are the mechanisms by which flavonoids and phenols exert antioxidant and anti-inflammatory effects [69].

Some studies suggest that *O. gratissimum* leaf extract proved to ameliorate the effects of lead acetate-induced cerebellar neurotoxicity in rats. It seems that decreasing of lipid peroxidation, reducing the levels of MDA, and increasing the activities of SOD, CAT, and GSH in the cerebellum repairs the integrity of cerebellum cells [190,191].

For *O. basilicum*, particularly its polyphenolic molecules, several preclinical studies have demonstrated their strong antioxidant [192,193,194,195,196,197,198,199] and neuroprotective effects [200,201]. The major and the most active compound isolated from *O. basilicum* is rosmarinic acid, a polyphenolic molecule with strong antioxidant activity [202]. This compound is considered the main component responsible for the neuroprotective effect. Although different mechanisms have been suggested, further studies are required to better understand its neuroprotective role. The synergistic action of various bioactive compounds in *O. basilicum* may further enhance its neuroprotective effects, making it a promising candidate for therapeutic applications in NDs [202].

The in vivo animal model studies have shown that scopolamine treatment can cause degradation of the hippocampal region, but two trimethoxy flavones from *O. basilicum* have improved the memory of animals, demonstrating neuroprotective action [65]. Many neuropsychological disorders such as AD can be caused by the negative effects of the harmful action of free radicals at the intracellular level.

Neuroprotective effects of methanolic extract of *O. basilicum* on an electromagnetic field-induced neurotoxicity model has been reported. The results of the study revealed an increase in SOD, GSH. and CAT activity, as well as a decrease in malondialdehyde (MDA) level, that proved the positive influence on oxidative damage in brain tissues. Also, in the studies regarding the activity of *O. basilicum* ethyl acetate extract in a global cerebral ischemia and reperfusion model, researchers observed the decrease in cerebral infarct size, lipid peroxidation and increase in GPx, and short-term memory and motor coordination [200,203].

Linalool is a monoterpene tertiary alcohol, a key compound of many essential oils from aromatic plants, including *O. basilicum*, which has demonstrated neuroprotective effects in animal models, effects associated with antioxidant and anti-inflammatory action [172].

The neuroprotective effect of *O. basilicum* and *O. gratissimum* has been demonstrated in vivo, as administration of extract of basil leaves, at doses of 200 and 400 mg/kg b.w., and improved scopolamine-induced memory deficit in mice. Furthermore, basil extracts had an antioxidant effect as well as inhibitory activity on AChE, resulting in a decrease in Ach synthesis and affecting cholinergic neurotransmission at the central level. This disruption of the cholinergic system is one of the mechanisms responsible for cognitive dysfunction in memory impairment as in AD [55,65,204].

Other investigations have highlighted the neuroprotective potential of *O. africanum* in experimental models of cognitive impairment. In a pivotal study employing scopolamine-induced memory deficits in rodents, administration of *O. africanum* volatile oil significantly increased latency time in the passive avoidance test, indicating improved memory retention. Concurrently, a notable reduction in AChE activity within brain tissue was observed, suggesting enhancement of central cholinergic neurotransmission, an effect associated with improved cognitive performance [200,203].

Recent data from the literature regarding the effects of *Ocimum* sp. in neurodegeneration, especially AD, is presented in Table 3, Table 4 and Table 5.

Even though clinical trials regarding the beneficial effects of *Ocimum* sp. are increasing, further research into its efficacy in neurodegenerative diseases is required. Jamshidi and Cohen have highlighted that a series of clinical studies using *Ocimum* sp. reported improvements in the participants’ mood, cognitive function, regardless of aspects such as gender, sex, formulation, or dose [205].

The neuroprotective, cognition-enhancing and stress-relieving effects of OS have been studied by Sampath et al., in a placebo-controlled clinical trial. In the study, 70% ethanolic extract of *Ocimum sanctum* (EtOS) was used, given as capsules (300 mg/day) to 40 healthy participants, for 4 weeks. The authors have highlighted the beneficial effects of EtOS compared to placebo on short-term memory, central executive functions of cognition such as information processing and retrieval, cognitive flexibility, and attention [206].

Another randomized, placebo-controlled study conducted by Saxenta et al. has reported positive findings. A total of 158 participants received capsules of OciBest (extract of the whole plant of *O. tenuiflorum* which contains 400 mg of actives) in a total dose which corresponds to 1200 mg of actives, for a 2-week period. The administration of the extract determined a significant decrease in the intensity of forgetfulness to about one-third observed in the placebo group, while also providing effective relief from frequent feeling of fatigue, being 39% more effective in the management of stress symptoms, compared to the placebo group [207].

In another clinical trial conducted by Bhattacharyya et al. in which 35 participants received 500 mg of *O. sanctum* plant extract as capsules, twice a day for 60 days, the authors reported an improvement in attention as well as stress and anxiety. However, it should be stated as a limitation that this study had no placebo control [208].

In an 8-week randomized, double-blind, placebo-controlled study conducted by Lopresti et al., the administration of an *Ociumum tenuiflorum* extract (250 mg/day) determined important improvements in self-report measures of perceived stress and sleep quality, also reducing stress response after exposure to an acute stressor and reduced chronic cortisol excretion [209]. This is important, since psychological stress increases the activity of the hypothalamic–pituitary–adrenal axis and thus the level of glucocorticoid hormones and it may also determine structural and functional damage to the hippocampus, also influencing learning and memory processes [210]. Additionally, the association between sleep and cognitive function is widely acknowledged, duet to the role of sleep in learning, memory, and synaptic plasticity as well as waste clearance from the brain [211].

Pertinent to safety, the authors have reported no serious adverse events during the trials we have mentioned, suggesting that the extracts that were used are considered safe.

**Table 5:** The effects of other *Ocimum* sp. in neurodegeneration.

*Ocimum* Species	Disease Model and Species	Neuroprotective Mechanism	Neuroprotective Effect	References
*O. gratissimum*	Wistar rats with cerebral ischemia	Antioxidant activity → free radicals scavenging Anti-inflammatory effects → blocking enzyme systems involved in the inflammatory process	Reduces oxidative stress, neuronal protection Limits neuroinflammation Enhances cognitive performance	[69]
*O. gratissimum*	Lead acetate-induced cerebellar neurotoxicity in rats	↓ lipid peroxidation ↓ MDA level ↑ antioxidant defense → ↑ SOD, CAT and GSH levels in the cerebellum	Ameliorates neurotoxicity in the cerebellum	[190,191]
*O. gratissimum*	Mouse model of scopolamine-induced memory deficit	↓ of AChE activity in brain tissue → ↓ of Ach synthesis → affecting cholinergic neurotransmission at the central level	Protection of the hippocampus Ameliorates memory deficits	[55,65,204]
*O. africanum*	Mouse model of scopolamine-induced memory deficit	Inhibits AChE activity in brain tissue → ↑ Ach activity Antioxidant activity → ↓ oxidative stress Improves performance in learning/memory behavioral tests	Improves learning and memory deficits Neuroprotective effects against oxidative damage	[200,203,212]
*O. americanum*	Wistar rats	Antioxidant activity → upregulates SOD, CAT, GSH Modulates cholinergic pathways	Neuroprotective effects in cognitive impairment models	[103,126]
		Anti-inflammatory activity	Suppresses neural inflammation	[126,213]
*O. kilimandscharicum*	BCCAO-induced cerebral ischemia-reperfusion injury in Swiss Albino mice	Antioxidant activity Modulation of neurotransmitter systems	Improved cognition/motor skills Reduced infarct size Enhanced antioxidant defenses	[200,214]

Legend: ↑, increase; ↓, decrease; →, results in.

## 9. Comparative Analysis of Phytochemical Profile and Neuroprotective Actions of *Ocimum* Species

The six species studied (*O. sanctum*, *O. basilicum*, *O. gratissimum*, *O. africanum*, *O. americanum*, and *O. kilimandscharicum*) have distinct phytochemical profiles and varying neuroprotective effects. The neuroprotective effects of *O. sanctum* are mainly attributed to eugenol, rosmarinic acid, orientin, and vicenin. It exhibits antioxidant effects and cholinergic upregulation (↑ChAT activity, ↓AChE) in various models of AD and ischemia. Significantly, *O. sanctum* diminishes Aβ and tau damage in rat models of AD [171,172,180].

*O. basilicum* is rich in rosmarinic acid, linalool, and methyl chavicol, that confers significant antioxidant protection (↓MDA, ↑SOD, ↑CAT, and ↑GPx), but a relatively weaker direct cholinergic modulation than *O. sanctum* [170]. Experimental evidence demonstrates that post-ischemic treatment with plant leaf extract results in marked improvements in memory and motor coordination, as well as a significant reduction in cerebral infarct size [215,216].

*O. gratissimum* contains elevated levels of caffeic acid, ellagic acid, and eugenol [217]. It is characterized by antioxidant and anti-inflammatory properties, resulting in inhibition of LOX/COX and NO generation. In the test of lead acetate-induced cerebellar neurotoxicity in rats, it ameliorates neurotoxicity in the cerebellum [190,191].

*O. africanum* demonstrates significant anti-AChE activity, indicating its potential for memory preservation in scopolamine-induced memory deficit models [212]. *O. americanum* has been reported to contain chlorogenic acid and rosmarinic acid, with demonstrated potent anti-AChE activity according to multiple phytochemical and pharmacological studies [50,143]. Comparative analyses indicate that *O. americanum* has greater cholinesterase inhibition than *O. basilicum*, evidenced by lower IC_50_ values and enhanced anti-AChE activity in both methanolic and essential oil extracts [66].

Regarding *O. kilimandscharicum*, it is abundant in camphor and borneol. The extracts have been subjected to limited neuropharmacological research. Nonetheless, preliminary investigations show that extracts of *O. kilimandscharicum* leaf can augment antioxidant defenses and reduce ischemia/reperfusion-induced brain injury in murine models [214].

Various chemical classes within the *Ocimum* sp. have distinct contributions to neuroprotection. Phenolic acids (rosmarinic acid, caffeic acid, and chlorogenic acid) are potent ROS scavengers, lipid peroxidation inhibitors, and metal chelators. Notably, rosmarinic acid has been shown to diminish Aβ aggregation in vitro, directly inhibiting the formation of toxic Aβ oligomers and fibrils through its binding to the β-sheet structure [218].

Flavonoids (apigenin, luteolin, orientin, vicenin, and quercetin) exert antioxidant properties, and also modulate key neurobiological signaling and inflammatory pathways relevant to neurodegenerative disease, such as PI3K/Akt and MAPK (including ERK1/2 and p38) [219].

Terpenoids and essential oils (eugenol, linalool, carvacrol, and borneol) demonstrate notable radical scavenging activity and directly influence neurotransmitter systems; for instance, eugenol potently inhibits AChE and MAO-B and also shows anti-aggregation activity against Aβ [220,221]. Linalool exerts anxiolytic and anti-inflammatory abilities primarily by modulating the GABAergic pathway [222]. Triterpenoids from *Ocimum* sp., specifically ursolic acid and oleanolic acid, are recognized for their ability to cross BBB and exhibit anti-inflammatory and anti-apoptotic properties [223].

## 10. Conclusions

The antioxidant and neuroprotective effects of different species of *Ocimum*—particularly *O. sanctum* and *O. basilicum*—are mainly attributed to their rich content of flavonoids, phenolic compounds, and essential oils. These bioactive compounds act synergistically to improve endogenous antioxidant enzyme activity, scavenge free radicals, and inhibit lipid peroxidation, thus protecting neuronal cells from oxidative damage and supporting cognitive health.

Notably, these plants have demonstrated therapeutic potential in different cellular and animal models of neurodegenerative disorders including AD, in which they have been shown to improve cognitive deficits and reduce pathological characteristic markers such as tau protein and beta-amyloid accumulation. Their efficacy was also highlighted in a number of clinical studies, in which they were shown to improve cognitive processes.

Nonetheless, further research is necessary in order to clarify the precise molecular mechanisms involved and to explore their potential as therapeutic agents in neurodegenerative diseases. Specifically, further work should aim to conduct randomized, double-blind, placebo-controlled trials in adults with a certain degree of cognitive impairment, to address pharmacokinetics, and bioavailability as safety, as well as to quantify synergistic interactions among *Ocimum* active compounds. This integrated approach will highlight the beneficial effects of *Ocimum* based interventions on neurodegenerative disorders, such as Alzheimer’s diseases.

## Figures and Tables

**Table 1:** Phytochemical composition of *Ocimum* species with neuroprotective effects.

Phytochemical Class	Identified Natural Compound	*Ocimum* Species	Relative Abundance/Supplementary References	References
Alcohols	Chlorogenic acid	*O. gratissimum*	+ [68]	[50,51,69]
		*O. basilicum*	++ [70]	
		*O. canum*	+	
		*O. kilimandscharicum*	+	
		*O. sanctum*	+ [71]	
		*O. citriodorum*	N/A	
Fatty acids	Linoleic acid	*O. sanctum* *O. americanum*	++ [72]	[50,55]
			++ [73]	
	Ocimumosides	*O. sanctum*	+	[49,62,74]
Flavonoids	Apigenin	*O. gratissimum* *O. sanctum* *O. basilicum* *O. citriodorum* *O. canum*	++	[50,58,69,75]
			++	
			+ [76]	
			+	
			+	
	Apigenin-7-O-glucuronide	*O. gratissimum* *O. basilicum* *O. canum* *O. kilimandscharicum* *O. sanctum* *O. citriodorum*	+/++ [52]	[49,51]
			+/++ [77]	
			+	
			+ [52]	
			++/+++ [52]	
			+ [52]	
	Catechin	*O. basilicum* *O. sanctum*	++ [78,79]	[80,81]
			+++ [80,82]	
	Cirsilineol Cirsimaritin	*O. americanum* *O. sanctum*	++ [83]	[50,58,74]
			+++	
	Isothymusin and isothymonin	*O. sanctum*	++ [84,85]	[50,58,74]
	Luteolin	*O. sanctum* *O. gratissimum* *O. citriodorum* *O. canum* *O. kilimandscharicum*	+ [86]	[49,50,51,69]
			++ [87]	
			++ [78]	
			N/A	
			N/A	
	Luteolin-7-O-glucuronide	*O. gratissimum* *O. basilicum* *O. kilimandscharicum* *O. sanctum*	++ [88]	[49,50,51]
			+ [52]	
			N/A [52]	
			+ [89]	
	Naringenin	*O. basilicum* *O. citriodorum*	+ [78,90]	[78,91]
			+ [78]	
	Rutin	*O. americanum* *O. basilicum* *O. campechianum* *O. citriodorum* *O. gratissimum* *O. kilimandscharicum* *O. selloi* *O. sanctum* *O. minimum* *O. africanum*	+	[50,66,69]
			++ [81]	
			++ [92]	
			+	
			++	
			+	
			N/A	
			+	
			+	
			+	
	Orientin (8-C glucoside of luteolin)	*O. sanctum*	++/+++ [93,94]	[50,51,55,95]
	Quercetin/isoquercetin	*O. gratissimum* *O. minimum* *O. africanum* *O. basilicum* *O. americanum*	++ [62,68]	[50,66]
			+ [96]	
			+ [97,98]	
			+ [96,98]	
			+ [96]	
	Salvigenin	*O. sanctum* *O. gratissimum*	+ [94]	[50,74]
			+ [69]	
	Vicenin	*O sanctum* *O. africanum* *O. minimum* *O. basilicum* *O. americanum*	++ [99,100,101,102]	[50,51,55,66]
			+ [96]	
			N/A [50,100]	
			+	
			+/0 * [50,100]	
	Vitexin	*O. americanum* *O. sanctum*	++ [103]	[50,51,74]
			+/N/A [50,62,104,105]	
Monoterpenes	Borneol	*O. basilicum* *O. americanum* *O. gratissimum* *O. sanctum*	++ [106]	[50,74,75]
			+/N/A [106,107]	
			+ [50,108]	
			+/N/A [106,107]	
	Carvacrol	*O. americanum* *O. gratissimum* *O. sanctum*	++/+++ [50,109]	[50,74]
			+/++ [50,108]	
			+ [110,111]	
	1,8-cineole, β-ocimene	*O. sanctum* *O. basilicum* *O. gratissimum* *O. campechianum*	+ [108,112]	[50,55,75]
			+/++ [112,113]	
			++ [50,114]	
			++ [92,112]	
	Linalool	*O. americanum* *O. basilicum* *O. campechianum* *O. sanctum*	++/+++ [115,116]	[50,64,74]
			+++ [116,117]	
			+++ [116]	
			++ [110]	
	β-pinene	*O. sanctum* *O. basilicum* *O. gratissimum* *O. campechianum*	+ [62,108,110,118]	[50,55,60]
			+ [106,113,119]	
			+ [69,108]	
			++ [120]	
Phenols	Caffeic acid	*O. basilicum* *O. sanctum* *O. gratissimum* *O. citriodorum* *O. americanum*	+++ [121,122]	[50,51,55,58]
			++ [51]	
			+ [68,123]	
			+ [124]	
			++ [125,126]	
	Chicoric acid	*O. americanum* *O. gratissimum*	+++ [127]	[50]
			++ [69,128]	
	Ellagic acid	*O. gratissimum* *O. americanum*	+ [69,129]	[50,69]
			N/A [52,130]	
	Eugenol	*O. sanctum* *O. basilicum* *O. americanum* *O. gratissimum* *O. campechianum* *O. kilimandscharicum* *O. sanctum*	+++ [72,86,110]	[49,50]
			++/+++ [113,131]	
			+ [132,133]	
			+++ [134,135]	
			+++ [45,112,116]	
			+++ [136,137]	
			+++ [86,114]	
	Ferulic acid	*O. sanctum*	+ [138]	[50,74]
	Gallic acid	*O. gratissimum*	+++ [62,139,140]	[69]
	Sinapic acid	*O. gratissimum* *O. sanctum*	+ [69,141] +/0 [142]	[50,69,74]
Phenylpropanoids	Rosmarinic acid	*O. americanum* *O. basilicum* *O. campechianum* *O. canum* *O. citriodorum* *O. gratissimum* *O. kilimandscharicum* *O. selloi* *O. sanctum*	+++ [52,143,144]	[49,50,58,69]
			+++ [52,145,146]	
			+++ [52,92]	
			++ [52,147]	
			+++ [52,124]	
			+++ [144,148,149]	
			++ [150,151]	
			+ [150,152]	
			+++ [51,153]	
	Methyl chavicol	*O. basilicum*	+++ [117,135,154]	[155]
	Methyl cinnamate	*O. basilicum*	+++ [117,156,157]	[50,64,75]
	Methyl eugenol	*O. sanctum* *O. campechianum* *O. gratissimum* *O. basilicum* *O. canum*	+++ [135,158]	[49,50,159]
			++/+++ [116,160]	
			+/0 [135,161]	
			+/++ [116]	
			N/A	
Sesquiterpenoids	Caryophyllene β-caryophyllene	*O. basilicum* *O. gratissimum* *O. campechianum* *O. sanctum*	++/+++ [113,131]	[50,51,74]
			++ [108,135]	
			++/+++ [92,112]	
			++ [110,158]	
Triterpenoids	Oleanolic acid	*O. gratissimum* *O. basilicum* *O. canum* *O. kilimandscharicum* *O. sanctum* *O. citriodorum*	++ [69,162,163]	[49,50,58,69]
			++ [50,164]	
			++ [165]	
			+/N/A [166]	
			++ [86]	
			N/A [124]	
	Ursolic acid	*O. sanctum*	+++ [167,168]	[50,74]

Legend: *, depending on variety; +, detected at low levels (~0.01–0.05 mg/g); ++, moderate levels (~0.05–0.2 mg/g); +++, high levels (>0.2 mg/g or one of the most abundant when compared to other compounds; N/A, quantitative data is unavailable; 0, absence is reported.

**Table 2:** Chemical structures of *Ocimum* species natural compounds with neuroprotective effects.

Phytochemical Class	Identified Natural Compound	Chemical Structure
Alcohols	Chlorogenic acid	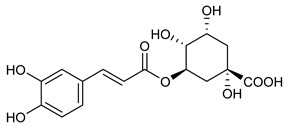
Fatty acids	Linoleic acid	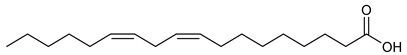
	Ocimumosides (e.g., type A)	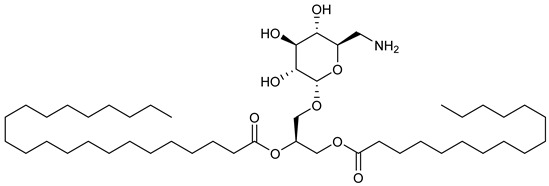
Flavonoids	Apigenin	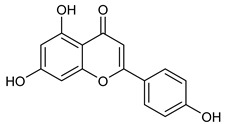
	Apigenin-7-O-glucuronide	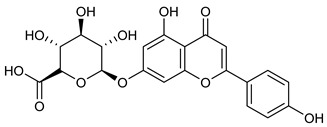
	Catechin	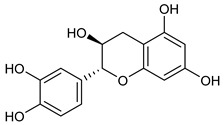
	Cirsilineol Cirsimaritin	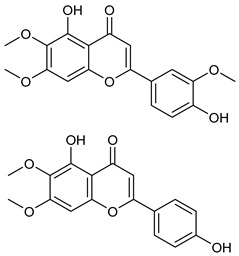
	Isothymusin and isothymonin	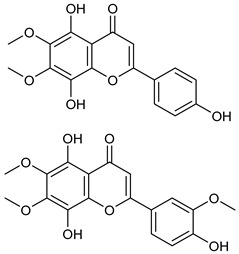
	Luteolin	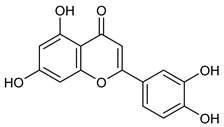
	Luteolin-7-O-glucuronide	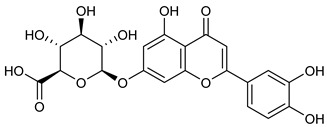
	Naringenin	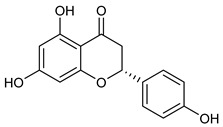
	Rutin	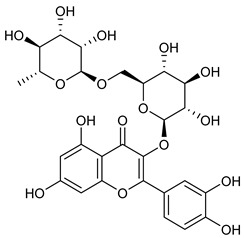
	Orientin (8-C glucoside of luteolin)	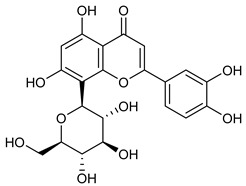
	Quercetin/isoquercetin	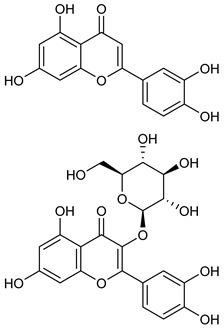
	Salvigenin	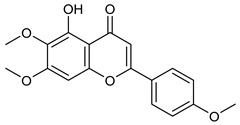
	Vicenin (vicenin 1)	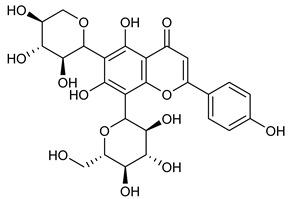
	Vitexin	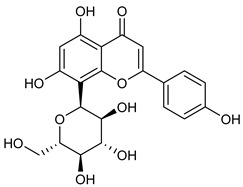
Monoterpenes	Borneol	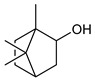
	Carvacrol	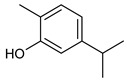
	1,8-cineole, β-ocimene	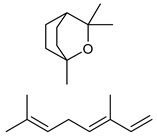
	Linalool	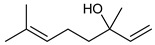
	β-pinene	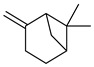
Phenols	Caffeic acid	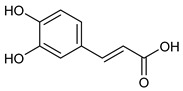
	Chicoric acid	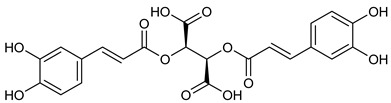
	Ellagic acid	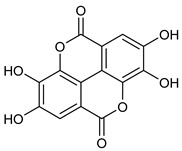
	Eugenol	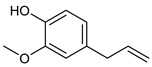
	Ferulic acid	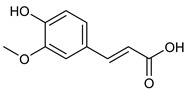
	Gallic acid	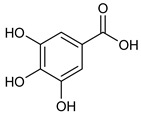
	Sinapic acid	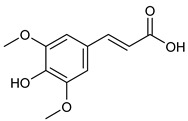
Phenylpropanoids	Rosmarinic acid	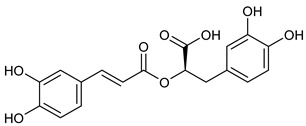
	Methyl chavicol	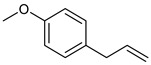
	Methyl cinnamate	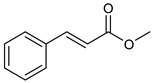
	Methyl eugenol	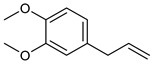
Sesquiterpenoids	Caryophyllene β-caryophyllene	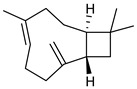
Triterpenoids	Oleanolic acid	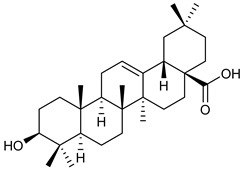
	Ursolic acid	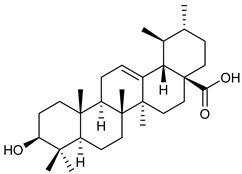

**Table 3:** The effects of *O. sanctum* in neurodegeneration.

*Ocimum* Species	Disease Model and Species	Neuroprotective Mechanism	Neuroprotective Effect	References
*O. sanctum* ethanolic extract	Rat model of noise stress induction	Prevents lipid peroxidation and oxidative stress	Neuroprotective properties against noise-induced oxidative stress	[55,56]
*O. sanctum* methanolic extract	Rat model of cerebral ischemia	Attenuates motor dysfunction Reduces the size of cerebral infarction	Improves cognitive function	[176]
*O. sanctum* hydroalcoholic extract	Wistar albino rats	Enhances the activity of endogenous antioxidant enzymes (SOD, CAT, GPx, GSH, and ascorbic acid)	Protects against cadmium-induced toxicity	[177]
*O. sanctum* extract and isolated flavonoids	Mouse liver	Significantly decreases MDA levels before γ-radiation	Reduces lipid peroxidation mediated through antioxidant effects	[178]
*O. sanctum* derived eugenol		Prevents of amyloid plaque formation and amyloid-induced hemolysis	Improves cognitive performance	[182,183]
	Rat liver mitochondria	Inhibits microsomal lipid peroxidation	Prevents oxidative damage and mitochondrial dysfunction in neurons	[55,184]
*O. sanctum* extract	Rat model of AD	↓ tau protein and beta-amyloid levels in the hippocampus Restores histological and neurochemical changes induced by Aβ Regulates neurotransmitter levels (the extract both alone and in combination with levetiracetam)	Restores neurochemical changes Improves cognitive function Reduces motor dysfunction Decrease the size of cerebral infarctions	[187]
*O. sanctum* extract	Rat model of cognitive dysfunction induced by administration of atropine, cyclosporine, or by maximal electroshock	↓ brain AChE activity → ↑ synaptic Ach	Improved cholinergic neurotransmission Improved cognitive performance	[188]
*O. sanctum* extract	Animal model	↑ ChAT activity → ↑ ACh synthesis	Behavioral improvement (radial maze performance) Enhance of memory function	[60]
*O. sanctum* ethanolic extract	Rat model of AD induced by TMT	↑ in the density of hippocampal neurons ↑ in neuropeptide expression	Preservation of neuronal structure and density Enhanced resistance of brain cells to neurotoxic factors exposure	[24]
*O. sanctum* extract	HEK-293 cells exposed to TMT	↑ of cell viability after TMT exposure Maintains ChAT expression	Anti-apoptotic activity Cell-protective role	[189]

Legend: ↑, increase; ↓, decrease; →, results in.

**Table 4:** The effects of *O. basilicum* in neurodegeneration.

*Ocimum* Species	Disease Model and Species	Neuroprotective Mechanism	Neuroprotective Effect	References
*O. basilicum* ethyl acetate extract	Global cerebral ischemia and reperfusion model	↓ in cerebral infarct size ↓ lipid peroxidation in brain tissue ↑ GPx activity	Improves short-term memory and motor coordination	[200,203]
*O. basilicum* methanolic extract	Electromagnetic field-induced neurotoxicity model	↑ levels of antioxidant enzymes (SOD, GSH, CAT) ↓ levels of MDA (marker of lipid peroxidation)	Positive influence on oxidative damage in brain tissues	[200,203]
*O. basilicum*	Mouse model of scopolamine-induced memory deficit	↓ of AChE activity in brain tissue → ↓ of ACh synthesis → affecting cholinergic neurotransmission at the central level	Protects the hippocampus Ameliorates memory deficits	[55,65,204]
*O. basilicum*		Antioxidant action AChE inhibition → ↓ ACh synthesis	Reduces neuronal damage Supports cognitive function, memory Counteracts memory impairment	[55,65,204]

Legend: ↑, increase; ↓, decrease; →, results in.

## Data Availability

No new data were created or analyzed in this study. Data sharing is not applicable to this article.

## Abbreviations

The following abbreviations are used in this manuscript:

Ach	Acetylcholine
AchE	Acetylcholinesterase
AD	Alzheimer’s disease
ALS	Amyotrophic lateral sclerosis
APP	Amyloid precursor protein
Aβ	Β-Amyloid
BBB	Blood–brain barrier
BCCAO	Bilateral common carotid artery occlusion
BHT	Butylated hydroxy toluene
CAT	Catalase
cGMP	Cyclic GMP
ChAT	Choline acetyltransferase
Cu^+^	Copper
ERK	Extracellular signal-regulated kinase
FMN	Flavin mononucleotide
GAE	Gallic acid equivalent
GPx	Glutathione peroxidase
GSH	Reduced glutathione
H_2_O_2_	Hydrogen peroxide
HD	Huntington’s disease
HEK-293	Human embryonic kidney-293
HO•	Hydroxyl radical
LPx	Lipid peroxidation
MAO	Monoamine oxidase
MDA	Malondialdehyde
MPTP	1-methyl-4-phenyl-1,2,3,6-tetrahydropyridine
MTDL	Multitarget-directed ligand
NADH	Nicotinamide adenine dinucleotide reduced
ND	Neurodegenerative disease
NO	Nitric oxide
*O. basilicum*	*Ocimum basilicum*
*O. sanctum*	*Ocimum sanctum*
O_2_^−^	Superoxide anion
ONOO^−^	Peroxynitrite
PARP	Poly (ADP-ribose) polymerase
PD	Parkinson’s disease
PGC-1α	Peroxisome proliferator-activated receptor gamma coactivator-1α
PKC	Protein kinase C
PUFA	Polyunsaturated fatty acid
QE	Quercetin equivalent
RNS	Reactive nitrogen species
ROS	Reactive oxygen species
SOD	Superoxide dismutase
TBHQ	Tert-butyl hydroquinone
TMT	Trimethyltin
Zn^2+^	Zinc

## References

[1] Dugger B.N., Dickson D.W. (2017). Pathology of Neurodegenerative Diseases. Cold Spring Harb. Perspect. Biol..

[2] Feigin V.L., Abajobir A.A., Abate K.H., Abd-Allah F., Abdulle A.M., Abera S.F., Abyu G.Y., Ahmed M.B., Aichour A.N., Aichour I. (2017). Global, regional, and national burden of neurological disorders during 1990-2015: A systematic analysis for the Global Burden of Disease Study 2015. Lancet Neurol..

[3] Goodfellow M.J., Borcar A., Proctor J.L., Greco T., Rosenthal R.E., Fiskum G. (2020). Transcriptional activation of antioxidant gene expression by Nrf2 protects against mitochondrial dysfunction and neuronal death associated with acute and chronic neurodegeneration. Exp. Neurol..

[4] Lebel M., Picard F., Ferland G., Gaudreau P. (2012). Drugs, nutrients, and phytoactive principles improving the health span of rodent models of human age-related diseases. J. Gerontol. A Biol. Sci. Med. Sci..

[5] Viña J., Lloret A., Ortí R., Alonso D. (2004). Molecular bases of the treatment of Alzheimer’s disease with antioxidants: Prevention of oxidative stress. Mol. Asp. Med..

[6] Rodrigo R., Miranda A., Vergara L. (2011). Modulation of endogenous antioxidant system by wine polyphenols in human disease. Clin. Chim. Acta.

[7] Melo A., Monteiro L., Lima R.M., Oliveira D.M., Cerqueira M.D., El-Bachá R.S. (2011). Oxidative stress in neurodegenerative diseases: Mechanisms and therapeutic perspectives. Oxid. Med. Cell Longev..

[8] Castellani R.J., Rolston R.K., Smith M.A. (2010). Alzheimer disease. Dis. Mon..

[9] Lee S.J., Koh J.Y. (2010). Roles of zinc and metallothionein-3 in oxidative stress-induced lysosomal dysfunction, cell death, and autophagy in neurons and astrocytes. Mol. Brain.

[10] Tarozzi A. (2020). Oxidative Stress in Neurodegenerative Diseases: From Preclinical Studies to Clinical Applications. J. Clin. Med..

[11] Uttara B., Singh A.V., Zamboni P., Mahajan R.T. (2009). Oxidative stress and neurodegenerative diseases: A review of upstream and downstream antioxidant therapeutic options. Curr. Neuropharmacol..

[12] Sikder M.M., Li X., Akumwami S., Labony S.A. (2025). Reactive Oxygen Species: Role in Pathophysiology, and Mechanism of Endogenous and Dietary Antioxidants during Oxidative Stress. Chonnam Med. J..

[13] Liguori I., Russo G., Curcio F., Bulli G., Aran L., Della-Morte D., Gargiulo G., Testa G., Cacciatore F., Bonaduce D. (2018). Oxidative stress, aging, and diseases. Clin. Interv. Aging.

[14] De la Fuente M., Miquel J. (2009). An update of the oxidation-inflammation theory of aging: The involvement of the immune system in oxi-inflamm-aging. Curr. Pharm. Des..

[15] Piantadosi C.A., Suliman H.B. (2012). Transcriptional control of mitochondrial biogenesis and its interface with inflammatory processes. Biochim. Biophys. Acta.

[16] Morroni F., Sita G., Graziosi A., Turrini E., Fimognari C., Tarozzi A., Hrelia P. (2018). Protective Effects of 6-(Methylsulfinyl)hexyl Isothiocyanate on Aβ(1-42)-Induced Cognitive Deficit, Oxidative Stress, Inflammation, and Apoptosis in Mice. Int. J. Mol. Sci..

[17] Morroni F., Sita G., Tarozzi A., Rimondini R., Hrelia P. (2016). Early effects of Aβ1-42 oligomers injection in mice: Involvement of PI3K/Akt/GSK3 and MAPK/ERK1/2 pathways. Behav. Brain Res..

[18] Awasthi M., Singh S., Pandey V.P., Dwivedi U.N. (2016). Alzheimer’s disease: An overview of amyloid beta dependent pathogenesis and its therapeutic implications along with in silico approaches emphasizing the role of natural products. J. Neurol. Sci..

[19] de Rus Jacquet A., Tambe M.A., Rochet J.-C., Coulston A.M., Boushey C.J., Ferruzzi M.G., Delahanty L.M. (2017). Chapter 18—Dietary Phytochemicals in Neurodegenerative Disease. Nutrition in the Prevention and Treatment of Disease.

[20] Kannappan R., Gupta S.C., Kim J.H., Reuter S., Aggarwal B.B. (2011). Neuroprotection by spice-derived nutraceuticals: You are what you eat!. Mol. Neurobiol..

[21] Nisbet R.M., Polanco J.C., Ittner L.M., Götz J. (2015). Tau aggregation and its interplay with amyloid-β. Acta Neuropathol..

[22] Preethi Pallavi M.C., Sampath Kumar H.M., Brahmachari G. (2018). Chapter 8—Nutraceuticals in Prophylaxis and Therapy of Neurodegenerative Diseases. Discovery and Development of Neuroprotective Agents from Natural Products.

[23] Kavitha R.V., Kumar J.R., Egbuna C., Ifemeje J.C., Egbuna C., Kumar S., Ifemeje J.C., Ezzat S.M., Kaliyaperumal S. (2020). Chapter 10—Phytochemicals as therapeutic interventions in neurodegenerative diseases. Phytochemicals as Lead Compounds for New Drug Discovery.

[24] Mataram M.B.A., Hening P., Harjanti F.N., Karnati S., Wasityastuti W., Nugrahaningsih D.A.A., Kusindarta D.L., Wihadmadyatami H. (2021). The neuroprotective effect of ethanolic extract *Ocimum sanctum* Linn. in the regulation of neuronal density in hippocampus areas as a central autobiography memory on the rat model of Alzheimer’s disease. J. Chem. Neuroanat..

[25] Barnham K.J., Bush A.I. (2008). Metals in Alzheimer’s and Parkinson’s diseases. Curr. Opin. Chem. Biol..

[26] Aras M.A., Aizenman E. (2011). Redox regulation of intracellular zinc: Molecular signaling in the life and death of neurons. Antioxid. Redox Signal.

[27] Jomova K., Valko M. (2011). Advances in metal-induced oxidative stress and human disease. Toxicology.

[28] Frazzini V., Rockabrand E., Mocchegiani E., Sensi S.L. (2006). Oxidative stress and brain aging: Is zinc the link?. Biogerontology.

[29] Anand A., Patience A.A., Sharma N., Khurana N. (2017). The present and future of pharmacotherapy of Alzheimer’s disease: A comprehensive review. Eur. J. Pharmacol..

[30] Giridharan V.V., Thandavarayan R.A., Konishi T., Martin C.R., Preedy V.R. (2015). Chapter 98—*Ocimum sanctum* Linn. (Holy Basil) to Improve Cognition. Diet and Nutrition in Dementia and Cognitive Decline.

[31] Bhatia S., Rawal R., Sharma P., Singh T., Singh M., Singh V. (2022). Mitochondrial Dysfunction in Alzheimer’s Disease: Opportunities for Drug Development. Curr. Neuropharmacol..

[32] Stolp H.B., Dziegielewska K.M. (2009). Review: Role of developmental inflammation and blood-brain barrier dysfunction in neurodevelopmental and neurodegenerative diseases. Neuropathol. Appl. Neurobiol..

[33] Sharma K., Verma R., Kumar D., Nepovimova E., Kuča K., Kumar A., Raghuvanshi D., Dhalaria R., Puri S. (2022). Ethnomedicinal plants used for the treatment of neurodegenerative diseases in Himachal Pradesh, India in Western Himalaya. J. Ethnopharmacol..

[34] Quinn J., Montine T., Morrow J., Woodward W.R., Kulhanek D., Eckenstein F. (2003). Inflammation and cerebral amyloidosis are disconnected in an animal model of Alzheimer’s disease. J. Neuroimmunol..

[35] Nichols E., Steinmetz J.D., Vollset S.E., Fukutaki K., Chalek J., Abd-Allah F., Abdoli A., Abualhasan A., Abu-Gharbieh E., Akram T.T. (2022). Estimation of the global prevalence of dementia in 2019 and forecasted prevalence in 2050: An analysis for the Global Burden of Disease Study 2019. Lancet Public Health.

[36] (2025). 2025 Alzheimer’s disease facts and figures. Alzheimers Dement..

[37] Raghavendra M., Maiti R., Kumar S., Acharya S. (2009). Role of *Ocimum sanctum* in the experimental model of Alzheimer’s disease in rats. Int. J. Green Pharm..

[38] Hrelia P., Sita G., Ziche M., Ristori E., Marino A., Cordaro M., Molteni R., Spero V., Malaguti M., Morroni F. (2020). Common Protective Strategies in Neurodegenerative Disease: Focusing on Risk Factors to Target the Cellular Redox System. Oxid. Med. Cell Longev..

[39] Andrade S., Ramalho M.J., Loureiro J.A., Pereira M.D.C. (2019). Natural Compounds for Alzheimer’s Disease Therapy: A Systematic Review of Preclinical and Clinical Studies. Int. J. Mol. Sci..

[40] Homody A.-A., Abood J., Dheeb B. (2020). The synergistic effect of fungus filter *Aspergillus terreus* and aqueous extract of Fucus vesiculosus on some growth characteristics of the ocimum basilicum and its content of active substances. EurAsian J. Biosci..

[41] Misra R., Das G. (2015). *Ocimum kilimandscharicum* Guerke (Lamiaceae): A New Distributional Record for Peninsular India with Focus on its Economic Potential. Proc. Natl. Acad. Sci. India Sect. B Biol. Sci..

[42] Chowdhury T., Mandal A., Roy S.C., De Sarker D. (2017). Diversity of the genus *Ocimum* (Lamiaceae) through morpho-molecular (RAPD) and chemical (GC-MS) analysis. J. Genet. Eng. Biotechnol..

[43] Singh S., Lal R.K., Maurya R., Chanotiya C.S. (2018). Genetic diversity and chemotype selection in genus *Ocimum*. J. Appl. Res. Med. Aromat. Plants.

[44] Anonymous The Plant List. Version 1.1—A Working List of All Plant Species. http://www.theplantlist.org/.

[45] Pandey A.K., Singh P., Tripathi N.N. (2014). Chemistry and bioactivities of essential oils of some *Ocimum* species: An overview. Asian Pac. J. Trop. Biomed..

[46] Carović-Stanko K., Liber Z., Besendorfer V., Javornik B., Bohanec B., Kolak I., Satovic Z. (2009). Genetic relations among basil taxa (*Ocimum* L.) based on molecular markers, nuclear DNA content, and chromosome number. Plant Syst. Evol..

[47] Bhagat M., Sangral M., Kumar A., Rather R.A., Arya K. (2020). Chemical, biological and in silico assessment of *Ocimum viride* essential oil. Heliyon.

[48] Zheljazkov V.D., Cantrell C.L., Tekwani B., Khan S.I. (2008). Content, composition, and bioactivity of the essential oils of three basil genotypes as a function of harvesting. J. Agric. Food Chem..

[49] Anusmitha K.M., Aruna M., Job J.T., Narayanankutty A., Pb B., Rajagopal R., Alfarhan A., Barcelo D. (2022). Phytochemical analysis, antioxidant, anti-inflammatory, anti-genotoxic, and anticancer activities of different *Ocimum* plant extracts prepared by ultrasound-assisted method. Physiol. Mol. Plant Pathol..

[50] Dharsono H.D.A., Putri S.A., Kurnia D., Dudi D., Satari M.H. (2022). *Ocimum* Species: A Review on Chemical Constituents and Antibacterial Activity. Molecules.

[51] Chaudhary A., Sharma S., Mittal A., Gupta S., Dua A. (2020). Phytochemical and antioxidant profiling of *Ocimum sanctum*. J. Food Sci. Technol..

[52] Beltrán-Noboa A., Jordan-Álvarez A., Guevara-Terán M., Gallo B., Berrueta L.A., Giampieri F., Battino M., Álvarez-Suarez J.M., Tejera E. (2023). Exploring the Chemistry of *Ocimum* Species Under Specific Extractions and Chromatographic Methods: A Systematic Review. ACS Omega.

[53] Zheng Z., Li Y., Jin G., Huang T., Zou M., Duan S. (2020). The biological role of arachidonic acid 12-lipoxygenase (ALOX12) in various human diseases. Biomed. Pharmacother..

[54] Kustiati U., Wihadmadyatami H., Kusindarta D.L. (2022). Dataset of Phytochemical and secondary metabolite profiling of holy basil leaf (*Ocimum sanctum* Linn) ethanolic extract using spectrophotometry, thin layer chromatography, Fourier transform infrared spectroscopy, and nuclear magnetic resonance. Data Brief.

[55] Pradhan D., Biswasroy P., Haldar J., Cheruvanachari P., Dubey D., Rai V.K., Kar B., Kar D.M., Rath G., Ghosh G. (2022). A comprehensive review on phytochemistry, molecular pharmacology, clinical and translational outfit of *Ocimum sanctum* L.. S. Afr. J. Bot..

[56] Samson J., Sheeladevi R., Ravindran R. (2007). Oxidative stress in brain and antioxidant activity of *Ocimum sanctum* in noise exposure. Neurotoxicology.

[57] Geetha R.K., Vasudevan D.M. (2004). Inhibition of lipid peroxidation by botanical extracts of *Ocimum sanctum*: In vivo and in vitro studies. Life Sci..

[58] Mahajan N., Rawal S., Verma M., Poddar M., Alok S. (2013). A phytopharmacological overview on *Ocimum* species with special emphasis on *Ocimum sanctum*. Biomed. Prev. Nutr..

[59] Joshi H., Parle M. (2006). Cholinergic basis of memory improving effect of *Ocimum tenuiflorum* Linn. Indian J. Pharm. Sci..

[60] Kusindarta D.L., Wihadmadyatami H., Jadi A.R., Karnati S., Lochnit G., Hening P., Haryanto A., Auriva M.B., Purwaningrum M. (2018). Ethanolic extract *Ocimum sanctum*. Enhances cognitive ability from young adulthood to middle aged mediated by increasing choline acetyl transferase activity in rat model. Res. Vet. Sci..

[61] Ravindran R., Rathinasamy S.D., Samson J., Senthilvelan M. (2005). Noise-stress-induced brain neurotransmitter changes and the effect of *Ocimum sanctum* (Linn) treatment in albino rats. J. Pharmacol. Sci..

[62] Venuprasad M.P., Kandikattu H.K., Razack S., Amruta N., Khanum F. (2017). Chemical composition of *Ocimum sanctum* by LC-ESI-MS/MS analysis and its protective effects against smoke induced lung and neuronal tissue damage in rats. Biomed. Pharmacother..

[63] Mohammed A.B.A., Yagi S., Tzanova T., Schohn H., Abdelgadir H., Stefanucci A., Mollica A., Mahomoodally M.F., Adlan T.A., Zengin G. (2020). Chemical profile, antiproliferative, antioxidant and enzyme inhibition activities of *Ocimum basilicum* L. and *Pulicaria undulata* (L.) C.A. Mey. grown in Sudan. S. Afr. J. Bot..

[64] Mahmoud E., Starowicz M., Ciska E., Topolska J., Farouk A. (2022). Determination of volatiles, antioxidant activity, and polyphenol content in the postharvest waste of *Ocimum basilicum* L.. Food Chem..

[65] Singh V., Kaur K., Kaur S., Shri R., Singh T.G., Singh M. (2022). Trimethoxyflavones from *Ocimum basilicum* L. leaves improve long term memory in mice by modulating multiple pathways. J. Ethnopharmacol..

[66] Farag M.A., Ezzat S.M., Salama M.M., Tadros M.G. (2016). Anti-acetylcholinesterase potential and metabolome classification of 4 *Ocimum* species as determined via UPLC/qTOF/MS and chemometric tools. J. Pharm. Biomed. Anal..

[67] Zhakipbekov K., Turgumbayeva A., Akhelova S., Bekmuratova K., Blinova O., Utegenova G., Shertaeva K., Sadykov N., Tastambek K., Saginbazarova A. (2024). Antimicrobial and Other Pharmacological Properties of *Ocimum basilicum*, *Lamiaceae*. Molecules.

[68] Kpètèhoto H.W., Amoussa A.M.O., Johnson R.C., Houéto E.E.M., Mignanwandé F.M.Z., Yédomonhan H., Loko F., Bankolé H., Lagnika L. (2019). Phytochemical analysis and antioxidant potential of *Ocimum gratissimum* Linn (*Lamiaceae*) commonly consumed in the Republic of Benin. J. Appl. Biol. Biotechnol..

[69] Ugbogu O.C., Emmanuel O., Agi G.O., Ibe C., Ekweogu C.N., Ude V.C., Uche M.E., Nnanna R.O., Ugbogu E.A. (2021). A review on the traditional uses, phytochemistry, and pharmacological activities of clove basil (*Ocimum gratissimum* L.). Heliyon.

[70] Mintas I., Tünde J., Gligor F., Craciun I., Luminita F., Patay E., Mureşan M., Udeanu D., Ionita A.-C., Antonescu A. (2019). Comparative phytochemical and antioxidative characterization of *Trifolium pratense* L.. and Ocimum basilicum L. Farmacia.

[71] Dixit H., Mathur P. (2023). Phytochemical Composition, Pharmacological Properties, and Therapeutic Applications of *Ocimum*. Curr. Trends Biotechnol. Microbiol..

[72] Pattanayak P., Behera P., Das D., Panda S.K. (2010). *Ocimum sanctum* Linn. A reservoir plant for therapeutic applications: An overview. Pharmacogn. Rev..

[73] Tironi de Castilho A.L., Costa Lima V.A., Pesse V.B., de Liori Teixeira L., Rozza A.L., Amalraj A., Kuttappan S., Varma A.C.K., Matharu A. (2023). Chapter 5—*Ocimum sanctum*. Herbs, Spices and Their Roles in Nutraceuticals and Functional Foods.

[74] Bhattarai K., Bhattarai R., Pandey R.D., Paudel B., Bhattarai H.D. (2024). A Comprehensive Review of the Phytochemical Constituents and Bioactivities of *Ocimum tenuiflorum*. Sci. World J..

[75] Du P., Yuan H., Chen Y., Zhou H., Zhang Y., Huang M., Jiangfang Y., Su R., Chen Q., Lai J. (2023). Identification of Key Aromatic Compounds in Basil (*Ocimum* L.) Using Sensory Evaluation, Metabolomics and Volatilomics Analysis. Metabolites.

[76] Wang M., Firrman J., Liu L., Yam K. (2019). A Review on Flavonoid Apigenin: Dietary Intake, ADME, Antimicrobial Effects, and Interactions with Human Gut Microbiota. Biomed. Res. Int..

[77] Kurnia D., Putri S.A., Tumilaar S.G., Zainuddin A., Dharsono H.D.A., Amin M.F. (2023). In silico Study of Antiviral Activity of Polyphenol Compounds from *Ocimum basilicum* by Molecular Docking, ADMET, and Drug-Likeness Analysis. Adv. Appl. Bioinform. Chem..

[78] Balanescu F., Mihaila M.D.I., Cârâc G., Furdui B., Vînătoru C., Avramescu S.M., Lisa E.L., Cudalbeanu M., Dinica R.M. (2020). Flavonoid Profiles of Two New Approved Romanian *Ocimum* Hybrids. Molecules.

[79] Qasem A., Assaggaf H., Mrabti H.N., Minshawi F., Rajab B.S., Attar A.A., Alyamani R.A., Hamed M., Mrabti N.N., Baaboua A.E. (2023). Determination of Chemical Composition and Investigation of Biological Activities of *Ocimum basilicum* L. *Molecules*
**2023**, *28*, 614. Molecules.

[80] Kondapalli N.B., Hemalatha R., Uppala S., Yathapu S.R., Mohammed S., Venkata Surekha M., Rajendran A., Bharadwaj D.K. (2022). *Ocimum sanctum*, Zingiber officinale, and Piper nigrum extracts and their effects on gut microbiota modulations (prebiotic potential), basal inflammatory markers and lipid levels: Oral supplementation study in healthy rats. Pharm. Biol..

[81] Nadeem H.R., Akhtar S., Sestili P., Ismail T., Neugart S., Qamar M., Esatbeyoglu T. (2022). Toxicity, Antioxidant Activity, and Phytochemicals of Basil (*Ocimum basilicum* L.) Leaves Cultivated in Southern Punjab, Pakistan. Foods.

[82] Ling A. (2009). Callus Induction of *Ocimum sanctum* and Estimation of Its Total Flavonoids Content. Asian J. Agric. Sci..

[83] Grayer R.J., Vieira R.F., Price A.M., Kite G.C., Simon J.E., Paton A.J. (2004). Characterization of cultivars within species of *Ocimum* by exudate flavonoid profiles. Biochem. Syst. Ecol..

[84] Utispan K., Niyomtham N., Yingyongnarongkul B.E., Koontongkaew S. (2020). Ethanolic Extract of Ocimum sanctum Leaves Reduced Invasion and Matrix Metalloproteinase Activity of Head and Neck Cancer Cell Lines. Asian Pac. J. Cancer Prev..

[85] Flegkas A., Milosević Ifantis T., Barda C., Samara P., Tsitsilonis O., Skaltsa H. (2019). Antiproliferative Activity of (-)-Rabdosiin Isolated from *Ocimum sanctum* L. *Medicines*
**2019**, *6*, 37. Medicines.

[86] Anandjiwala S., Kalola J., Rajani M. (2006). Quantification of eugenol, luteolin, ursolic acid, and oleanolic acid in black (*Krishna Tulasi*) and green (*Sri Tulasi*) varieties of *Ocimum sanctum* Linn. using high-performance thin-layer chromatography. J. AOAC Int..

[87] Irondi E.A., Agboola S.O., Oboh G., Boligon A.A. (2016). Inhibitory effect of leaves extracts of *Ocimum basilicum* and *Ocimum gratissimum* on two key enzymes involved in obesity and hypertension in vitro. J. Intercult. Ethnopharmacol..

[88] Zouine N., Ghachtouli N.E., Abed S.E., Koraichi S.I. (2024). A comprehensive review on medicinal plant extracts as antibacterial agents: Factors, mechanism insights and future prospects. Sci. Afr..

[89] Yadav A., Singh Y., Wal P., Srivastava A., Wal A., Srivastava P., Verma P., Kumar N., Sachan S. (2023). A Comprehensive Review on Phytoconstituents and Pharmacological Activity of Holy Basil *Ocimum sanctum* (Tulsi) Section. Eur. Chem. Bull..

[90] Teofilović B., Grujić-Letić N., Karadžić M., Kovačević S., Podunavac-Kuzmanović S., Gligorić E., Gadžurić S. (2021). Analysis of functional ingredients and composition of *Ocimum basilicum*. S. Afr. J. Bot..

[91] Hallmann E., Rusaczonek A., Muszyńska E., Ziółkowski D., Kuliński S., Jasek J., Ponder A. (2024). A Long-Term Study on Chemical Compounds and Their Location in Sweet Basil Leaves from Organic and Conventional Producers. Foods.

[92] Tacchini M., Echeverria Guevara M.P., Grandini A., Maresca I., Radice M., Angiolella L., Guerrini A. (2020). *Ocimum* campechianum Mill. from Amazonian Ecuador: Chemical Composition and Biological Activities of Extracts and Their Main Constituents (Eugenol and Rosmarinic Acid). Molecules.

[93] Lam K.Y., Ling A.P., Koh R.Y., Wong Y.P., Say Y.H. (2016). A Review on Medicinal Properties of Orientin. Adv. Pharmacol. Sci..

[94] Samanth M. (2025). The Chemical Constituents of Ocimum sanctum and Its Pharmacological Applications: A Review. Recent Developments in Chemistry and Biochemistry Research.

[95] Raza Ishaq A., El-Nashar H.A.S., Al-Qaaneh A.M., Asfandyar, Bashir A., Younis T. (2025). Orientin: A natural glycoside with versatile pharmacological activities. Nat. Prod. Res..

[96] Grayer R.J., Kite G.C., Veitch N.C., Eckert M.R., Marin P.D., Senanayake P., Paton A.J. (2002). Leaf flavonoid glycosides as chemosystematic characters in *Ocimum*. Biochem. Syst. Ecol..

[97] Yaldiz G., Camlica M. (2022). Essential oils content, composition and antioxidant activity of selected basil (*Ocimum basilicum* L.) genotypes. S. Afr. J. Bot..

[98] Rai A.K., Khan S., Kumar A., Dubey B.K., Lal R.K., Tiwari A., Trivedi P.K., Elliott C.T., Ch R. (2023). Comprehensive Metabolomic Fingerprinting Combined with Chemometrics Identifies Species- and Variety-Specific Variation of Medicinal Herbs: An *Ocimum* Study. Metabolites.

[99] Ali H., Dixit S. (2012). In Vitro antimicrobial activity of flavanoids of *Ocimum sanctum* with synergistic effect of their combined form. Asian Pac. J. Trop. Dis..

[100] Girme A., Bhoj P., Saste G., Pawar S., Mirgal A., Raut D., Chavan M., Hingorani L. (2021). Development and Validation of RP-HPLC Method for Vicenin-2, Orientin, Cynaroside, Betulinic Acid, Genistein, and Major Eight Bioactive Constituents with LC-ESI-MS/MS Profiling in *Ocimum* Genus. J. AOAC Int..

[101] Joseph B., Nair V.M. (2013). Ethanopharmacological and Phytochemical Aspects of *Ocimum sanctum* Linn—The Elixir of Life. J. Pharm. Res. Int..

[102] Li Y., Zheng Y., Wang H. (2021). Anticancer activity of Vicenin-2 against 7,12 dimethylbenz[a]anthracene-induced buccal pouch carcinoma in hamsters. J. Biochem. Mol. Toxicol..

[103] Siqueira I.R., Vanzella C., Lovatel G.A., Bertoldi K., Spindler C., Moysés F.d.S., Vizuete A., Poser G.L.v., Netto C.A. (2025). American Basil, *Ocimum americanum*, Has Neuroprotective Properties in the Aging Process. Nutrients.

[104] Arya R., Faruquee H.M., Shakya H., Rahman S.A., Begum M.M., Biswas S.K., Apu M.A.I., Islam M.A., Sheikh M.M.I., Kim J.J. (2024). Harnessing the Antibacterial, Anti-Diabetic and Anti-Carcinogenic Properties of *Ocimum sanctum* Linn (Tulsi). Plants.

[105] Priya S., Peddha M.S. (2023). Physicochemical characterization, polyphenols and flavonoids of different extracts from leaves of four varieties of tulsi (*Ocimum* sp.). S. Afr. J. Bot..

[106] IliĆ A.S., AntiĆ M.P., JelaČIĆ S.C., ŠOleviĆ Knudsen T.M. (2018). Chemical Composition of the Essential Oils of Three *Ocimum basilicum* L. Cultivars from Serbia. Not. Bot. Horti Agrobot. Cluj-Napoca.

[107] Gurav T.P., Dholakia B.B., Giri A.P. (2022). A glance at the chemodiversity of *Ocimum* species: Trends, implications, and strategies for the quality and yield improvement of essential oil. Phytochem. Rev..

[108] Tomar H., Rawat A., Nagarkoti K., Prakash O., Kumar R., Srivastava R.M., Rawat S., Rawat D.S. (2023). *Ocimum gratissimum* L. and *Ocimum sanctum* L.: Comparative compositional analysis of essential oils and in-vitro biological activities with in-silico PASS prediction and ADME/Tox studies. S. Afr. J. Bot..

[109] Sutili F.J., Velasquez A., Pinheiro C.G., Heinzmann B.M., Gatlin D.M., Baldisserotto B. (2016). Evaluation of *Ocimum americanum* essential oil as an additive in red drum (*Sciaenops ocellatus*) diets. Fish Shellfish. Immunol..

[110] Bhatnagar A., Pimoli R. (2025). Chemical composition of the essential oil of *Ocimum sanctum* L. growing in Garhwal region of Uttarakhand, India. Int. J. Herb. Med..

[111] Shanmugam K.R., Siva M., Ravi S., Shanmugam B., Reddy K.S. (2019). Bioactive Compound of *Ocimum sanctum* Carvacrol Supplementation Attenuates Fluoride Toxicity in Sodium Fluoride Intoxicated Rats: A Study with Respect to Clinical Aspect. Pharmacogn. Mag..

[112] Wilson T.M., Murphy B.J., Abad A., Packer C., Poulson A., Carlson R.E. (2022). Essential Oil Composition and Stable Isotope Profile of Cultivated *Ocimum campechianum* Mill. (*Lamiaceae*) from Peru. Molecules.

[113] ÖZcan M., Chalchat J.-C. (2002). Essential Oil Composition of *Ocimum basilicum* L. and *Ocimum minimum* L. in Turkey. Czech J. Food Sci..

[114] Padalia R.C., Verma R.S. (2011). Comparative volatile oil composition of four *Ocimum* species from northern India. Nat. Prod. Res..

[115] Vieira P.R.N., de Morais S.M., Bezerra F.H.Q., Travassos Ferreira P.A., Oliveira Í.R., Silva M.G.V. (2014). Chemical composition and antifungal activity of essential oils from *Ocimum* species. Ind. Crops Prod..

[116] Mohammed F., Uysal İ., Sevindik E., Sevindik M. (2023). Genus *Ocimum* in Terms of Mineral, Nutrient, Chemical Contents and Biological Activity. J. Microbiol. Biotechnol. Food Sci..

[117] Kholiya S., Punetha A., Cha uhan A., Kt V., Kumar D., Upadhyay R.K., Padalia R.C. (2022). Essential oil yield and composition of *Ocimum basilicum* L. at different phenological stages, plant density and post-harvest drying methods. S. Afr. J. Bot..

[118] Saharkhiz M.J., Kamyab A.A., Kazerani N.K., Zomorodian K., Pakshir K., Rahimi M.J. (2015). Chemical Compositions and Antimicrobial Activities of *Ocimum sanctum* L. Essential Oils at Different Harvest Stages. Jundishapur J. Microbiol..

[119] El-Soud N.H., Deabes M., El-Kassem L.A., Khalil M. (2015). Chemical Composition and Antifungal Activity of *Ocimum basilicum* L. Essential Oil. Open Access Maced. J. Med. Sci..

[120] Matasyoh L.G., Matasyoh J.C., Wachira F.N., Kinyua M.G., Muigai A.W., Mukiama T.K. (2008). Antimicrobial activity of essential oils of *Ocimum gratissimum* L. From different populations of Kenya. Afr. J. Tradit. Complement. Altern. Med..

[121] Romano R., De Luca L., Aiello A., Pagano R., Di Pierro P., Pizzolongo F., Masi P. (2022). Basil (*Ocimum basilicum* L.) Leaves as a Source of Bioactive Compounds. Foods.

[122] Bajomo E.M., Aing M.S., Ford L.S., Niemeyer E.D. (2022). Chemotyping of commercially available basil (*Ocimum basilicum* L.) varieties: Cultivar and morphotype influence phenolic acid composition and antioxidant properties. NFS J..

[123] Ye J.-C., Hsiao M.-W., Hsieh C.-H., Wu W.-C., Hung Y.-C., Chang W.-C. (2010). Analysis of Caffeic Acid Extraction From *Ocimum gratissimum* Linn. by High Performance Liquid Chromatography and its Effects on a Cervical Cancer Cell Line. Taiwan. J. Obstet. Gynecol..

[124] Majdi C., Pereira C., Dias M.I., Calhelha R.C., Alves M.J., Rhourri-Frih B., Charrouf Z., Barros L., Amaral J.S., Ferreira I. (2020). Phytochemical Characterization and Bioactive Properties of Cinnamon Basil (*Ocimum basilicum* cv. ‘Cinnamon’) and Lemon Basil (*Ocimum × citriodorum*). Antioxidants.

[125] Jasicka-Misiak I., Shanaida M., Hudz N., Wieczorek P.P. (2021). Phytochemical and Pharmacological Evaluation of the Residue By-Product Developed from the *Ocimum americanum* (*Lamiaceae*) Postdistillation Waste. Foods.

[126] Zengin G., Ferrante C., Gnapi D.E., Sinan K.I., Orlando G., Recinella L., Diuzheva A., Jekő J., Cziáky Z., Chiavaroli A. (2019). Comprehensive approaches on the chemical constituents and pharmacological properties of flowers and leaves of American basil (*Ocimum americanum* L). Food Res. Int..

[127] Lee J., Scagel C.F. (2009). Chicoric acid found in basil (*Ocimum basilicum* L.) leaves. Food Chem..

[128] Casanova L.M., da Silva D., Sola-Penna M., de Magalhães Camargo L.M., de Moura Celestrini D., Tinoco L.W., Costa S.S. (2014). Identification of chicoric acid as a hypoglycemic agent from *Ocimum gratissimum* leaf extract in a biomonitoring in vivo study. Fitoterapia.

[129] Ajayi A.M., Martins D.T.d.O., Balogun S.O., Oliveira R.G.d., Ascêncio S.D., Soares I.M., Barbosa R.d.S., Ademowo O.G. (2017). *Ocimum gratissimum* L. leaf flavonoid-rich fraction suppress LPS-induced inflammatory response in RAW 264.7 macrophages and peritonitis in mice. J. Ethnopharmacol..

[130] Green M., Pragada R.R., Ethadi S., Rajanna B. (2013). Comparative study on some selected species of & *Ocimum* genus on free radical scavenging activity and hepatoprotective activity against CCl4 induced intoxication in rats. Am. J. Mol. Biol..

[131] Hikmawanti N.P.E., Hariyanti H., Nurkamalia N., Nurhidayah S. (2019). Chemical Components of *Ocimum basilicum* L. and *Ocimum tenuiflorum* L. Stem Essential Oils and Evaluation of Their Antioxidant Activities Using DPPH Method. Pharm. Sci. Res..

[132] Tine Y., Antonio A., Sinzogan A.A.C., Ndiaye O., Sambou C., Diallo A., Mbenga I., Badji K., Hadji E., Elhadji D. (2024). The Essential Oil of *Ocimum americanum* from Senegal and Gambia as a Source of Methyleugenol for the Control of *Bactrocera dorsalis*, Fruit Fly. J. Agric. Chem. Environ..

[133] Hirose S., Sakai K., Kobayashi S., Tsuro M., Morikami A., Tsukagoshi H. (2024). Eugenol transport and biosynthesis through grafting in aromatic plants of the *Ocimum* genus. Plant Biotechnol..

[134] Ashokkumar K., Pandian A., Murugan M., Dhanya M.K., Vellaikumar S. (2021). Phytochemistry and pharmacological properties of *Ocimum gratissimum* (L.) extracts and essential oil—A critical review. J. Curr. Opin. Crop Sci..

[135] Joshi R.K. (2013). Chemical Composition, In Vitro Antimicrobial and Antioxidant Activities of the Essential Oils of *Ocimum Gratissimum*, *O. Sanctum* and their Major Constituents. Indian J. Pharm. Sci..

[136] Ayoubi R., Singh G., Pandey D. (2023). Chemical Composition of *Ocimum kilimandscharicum* Guerke Essential Oil Cultivated in Phagwara, Punjab, India. Hamdard Med..

[137] Ayoubi R., Wali S., Singh G.B. (2022). The UV and FTIR Fingerprint of *Ocimum kilimandscharicum* Guerke Essential oil: A Eugenol-Rich Chemo Type. Int. J. Innov. Res. Sci. Stud..

[138] Shanaida M.I., Cherevko M.O. (2024). Chromatographic analysis of flavonoids and phenolic acids in the herb *Ocimum sanctum* L.. Farmatsevtychnyi Zhurnal.

[139] Oriakhi K., Ehigbai O., Ezeugwu N., Ogechukwu A., Omorede A., Ehimwenma O. (2013). Comparative Antioxidant Activities of Extracts of Vernonia amygdalina and *Ocimum gratissimum* Leaves. J. Agric. Sci..

[140] Patel F., Modi N. (2018). Estimation of total phenolic content in selected varieties of *Ocimum* species grown in different environmental condition. J. Pharmacogn. Phytochem..

[141] Seal T., Ganguly S., Kumar J. (2021). Characterization of secondary metabolites in different parts of *Ocimum gratissimum* L. by In Vitro antioxidant activity and high-performance liquid chromatography–diode-array detector analysis. Pharmacogn. Mag..

[142] Ullah S., Rauf N., Hussain A., Sheikh I., Farooq M. (2022). HPLC profile of phenolic acids and flavonoids of *Ocimum sanctum* and *O. basilicum*. Int. J. Plant Based Pharm..

[143] Rady M.R., Nazif N.M. (2005). Rosmarinic acid content and RAPD analysis of in vitro regenerated basil (*Ocimum americanum*) plants. Fitoterapia.

[144] Tshilanda D., Kapepula P., Onyamboko D., Babady P., Tsalu P., Tshibangu D., Ngombe N., Frederich M., Ngbolua K.-T.-N., Mpiana P.T. (2016). Chemical Fingerprint and Anti-Sickling Activity of Rosmarinic Acid and Methanolic Extracts from Three Species of *Ocimum* from DR Congo. J. Biosci. Med..

[145] Touiss I., Harnafi M., Khatib S., Bekkouch O., Ouguerram K., Amrani S., Harnafi H. (2019). Rosmarinic acid-rich extract from *Ocimum basilicum* L. decreases hyperlipidemia in high fat diet-induced hyperlipidemic mice and prevents plasma lipid oxidation. Physiol. Pharmacol..

[146] Kwon D.Y., Li X., Kim J.K., Park S.U. (2019). Molecular cloning and characterization of rosmarinic acid biosynthetic genes and rosmarinic acid accumulation in *Ocimum basilicum* L.. Saudi J. Biol. Sci..

[147] Berhow M., Affum A., Gyan B. (2012). Rosmarinic Acid Content in Antidiabetic Aqueous Extract of *Ocimum canum* Sims Grown in Ghana. J. Med. Food.

[148] Costa R.S., Carneiro T.C.B., Cerqueira-Lima A.T., Queiroz N.V., Alcântara-Neves N.M., Pontes-de-Carvalho L.C., Velozo E.d.S., Oliveira E.J., Figueiredo C.A. (2012). *Ocimum gratissimum* Linn. and rosmarinic acid, attenuate eosinophilic airway inflammation in an experimental model of respiratory allergy to *Blomia tropicalis*. Int. Immunopharmacol..

[149] Prommajak T., Kim S.M., Pan C.-H., Kim S., Surawang S., Rattanapanone N. (2016). Identification of antioxidants in Lamiaceae vegetables by HPLC-ABTS and HPLC-MS. Chiang Mai Univ. J. Nat. Sci..

[150] Trakoontivakorn G., Tangkanakul P., Nakahara K. (2012). Changes of Antioxidant Capacity and Phenolics in *Ocimum* Herbs after Various Cooking Methods. Jpn. Agric. Res. Q..

[151] Agarwal K., Singh D., Jyotshna J., Ahmad A., Shanker K., Tandon S., Luqman S. (2017). Antioxidative potential of two chemically characterized *Ocimum* (Tulsi) species extracts. Biomed. Res. Ther..

[152] Alegría-Herrera E., Herrera-Ruiz M., Román-Ramos R., Zamilpa A., Santillán-Urquiza M.A., Aguilar M.I., Avilés-Flores M., Fuentes-Mata M., Jiménez-Ferrer E. (2019). Effect of *Ocimum basilicum*, *Ocimum selloi*, and Rosmarinic Acid on Cerebral Vascular Damage in a Chronic Hypertension Model. Biol. Pharm. Bull..

[153] Giraldo L., Castro V., Gregory F., Dias A.C.P. (2014). Potential of *Ocimum sanctum* L. cell suspensions for rosmarinic acid production. Planta Med..

[154] Padalia R.C., Verma R.S., Upadhyay R.K., Chauhan A., Singh V.R. (2017). Productivity and essential oil quality assessment of promising accessions of *Ocimum basilicum* L. from north India. Ind. Crops Prod..

[155] Lewinsohn E., Ziv-Raz I., Dudai N., Tadmor Y., Lastochkin E., Larkov O., Chaimovitsh D., Ravid U., Putievsky E., Pichersky E. (2000). Biosynthesis of estragole and methyl-eugenol in sweet basil (*Ocimum basilicum* L). Developmental and chemotypic association of allylphenol O-methyltransferase activities. Plant Sci..

[156] Morsy N.F.S., Hammad K.S.M. (2021). Extraction of essential oil from methyl cinnamate basil (*Ocimum canum* Sims) with high yield in a short time using enzyme pretreatment. J. Food Sci. Technol..

[157] Klimánková E., Holadová K., Hajšlová J., Čajka T., Poustka J., Koudela M. (2008). Aroma profiles of five basil (*Ocimum basilicum* L.) cultivars grown under conventional and organic conditions. Food Chem..

[158] Joshi D.R.K., Sharma A. (2021). Determination of Seasonal Variation of Volatile Organic Constituents of the Leaves of Traditional Herb *Ocimum sanctum* Linn. Indian J. Pharm. Sci..

[159] Mohamed A.E., Shetta A., Kegere J., Mamdouh W. (2022). Antibacterial and antioxidant properties of *Cichorium intybus* extract embedded in chitosan nanocomposite nanofibers. Int. J. Biol. Macromol..

[160] Pino Benitez N., Meléndez León E.M., Stashenko E.E. (2009). Eugenol and methyl eugenol chemotypes of essential oil of species *Ocimum gratissimum* L. and *Ocimum campechianum* Mill. from Colombia. J. Chromatogr. Sci..

[161] Thounaojam A.S., Sakure A.A., Dhaduk H.L., Kumar S., Mistry J.G. (2020). Impact evaluation of growth stage and species on morpho-physiological traits and bioactive constituent of essential oil in *Ocimum* through multi-year experiment. Ind. Crops Prod..

[162] Nganteng D.N.D., Melong R., Mbiekop E.P., Maffo T., Allémann É., Delie F., Wafo P., Tchaleu B.N., Dzoyem J.P. (2022). Chemical constituents and cytotoxic activity of *Ocimum gratissimum* L.. S. Afr. J. Bot..

[163] Njoku C.J., Zeng L., Asuzu I.U., Oberlies N.H., McLaughlin J.L. (1997). Oleanolic Acid, a Bioactive Component of the Leaves of *Ocimum gratissimum* (Lamiaceae). Int. J. Pharmacogn..

[164] Misra R.C., Sharma S., Sandeep, Garg A., Chanotiya C.S., Ghosh S. (2017). Two CYP716A subfamily cytochrome P450 monooxygenases of sweet basil play similar but nonredundant roles in ursane- and oleanane-type pentacyclic triterpene biosynthesis. New Phytol..

[165] Xaasan C.C., Cabdulraxmaan A.D., Passannanti S., Piozzi F., Schmid J.P. (1981). Constituents of the Essential Oil of *Ocimum canum*. J. Nat. Prod..

[166] Ghani S., Khan Z.H. (2015). Variation of Ursolic Acid Content in Four *Ocimum* Species from Four Different places of Maharashtra by HPTLC. World J. Pharm. Sci..

[167] Silva M.G., Vieira I.G., Mendes F.N., Albuquerque I.L., dos Santos R.N., Silva F.O., Morais S.M. (2008). Variation of ursolic acid content in eight *Ocimum* species from northeastern Brazil. Molecules.

[168] Vetal M.D., Lade V.G., Rathod V.K. (2012). Extraction of ursolic acid from *Ocimum sanctum* leaves: Kinetics and modeling. Food Bioprod. Process..

[169] Gandhi S., Abramov A.Y. (2012). Mechanism of oxidative stress in neurodegeneration. Oxid. Med. Cell Longev..

[170] Seyed M.A., Ayesha S., Azmi N., Al-Rabae F.M., Al-Alawy A.I., Al-Zahrani O.R., Hawsawi Y. (2021). The neuroprotective attribution of *Ocimum basilicum*: A review on the prevention and management of neurodegenerative disorders. Future J. Pharm. Sci..

[171] Cabezas R., Fidel Avila M., Torrente D., Gonzalez J., Santos El-Bachá R., Guedes R., Barreto G.E., Martin C.R., Preedy V.R. (2015). Chapter 76—Natural Antioxidants in Dementia: An Overview. Diet and Nutrition in Dementia and Cognitive Decline.

[172] Gonçalves S., Mansinhos I., Romano A., Martin C.R., Preedy V.R. (2020). Chapter 11—Aromatic plants: A source of compounds with antioxidant and neuroprotective effects. Oxidative Stress and Dietary Antioxidants in Neurological Diseases.

[173] Dini I., Holban A.M., Grumezescu A.M. (2018). Chapter 14—Spices and Herbs as Therapeutic Foods. Food Quality: Balancing Health and Disease.

[174] Makri O., and Kintzios S. (2008). *Ocimum* sp. (Basil): Botany, Cultivation, Pharmaceutical Properties, and Biotechnology. J. Herbs Spices Med. Plants.

[175] Kelm M.A., Nair M.G., Strasburg G.M., DeWitt D.L. (2000). Antioxidant and cyclooxygenase inhibitory phenolic compounds from *Ocimum sanctum* Linn. Phytomedicine.

[176] Yanpallewar S.U., Rai S., Kumar M., Acharya S.B. (2004). Evaluation of antioxidant and neuroprotective effect of *Ocimum sanctum* on transient cerebral ischemia and long-term cerebral hypoperfusion. Pharmacol. Biochem. Behav..

[177] Ramesh B., Satakopan V.N. (2010). Antioxidant Activities of Hydroalcoholic Extract of *Ocimum sanctum* Against Cadmium Induced Toxicity in Rats. Indian. J. Clin. Biochem..

[178] Uma Devi P., Ganasoundari A., Vrinda B., Srinivasan K.K., Unnikrishnan M.K. (2000). Radiation protection by the ocimum flavonoids orientin and vicenin: Mechanisms of action. Radiat. Res..

[179] Pandey G., Sharma M. (2010). Pharmacological activities of *Ocimum sanctum* (Tulsi): A review. Int. J. Pharm. Sci. Rev. Res..

[180] Singh D., Chaudhuri P.K. (2018). A review on phytochemical and pharmacological properties of Holy basil (*Ocimum sanctum* L.). Ind. Crops Prod..

[181] Patra J.K., Das G., Lee S., Kang S.-S., Shin H.-S. (2018). Selected commercial plants: A review of extraction and isolation of bioactive compounds and their pharmacological market value. Trends Food Sci. Technol..

[182] Jung M.J., Kim N., Jeon S.H., Gee M.S., Kim J.W., Lee J.K. (2023). Eugenol relieves the pathological manifestations of Alzheimer’s disease in 5×FAD mice. Phytomedicine.

[183] Taheri P., Yaghmaei P., Tehrani H.S., Ebrahim-Habibi A. (2019). Effects of Eugenol on Alzheimer’s Disease-like Manifestations in Insulin- and A[beta]-Induced Rat Models. Neurophysiology.

[184] Dubey K., Anand B.G., Shekhawat D.S., Kar K. (2017). Eugenol prevents amyloid formation of proteins and inhibits amyloid-induced hemolysis. Sci. Rep..

[185] Sharma A., Bhardwaj G., Sohal H.S., Gohain A., Kour J., Nayik G.A. (2022). Chapter 9—Eugenol. Nutraceuticals and Health Care.

[186] Barboza J.N., da Silva Maia Bezerra Filho C., Silva R.O., Medeiros J.V.R., de Sousa D.P. (2018). An Overview on the Anti-inflammatory Potential and Antioxidant Profile of Eugenol. Oxid. Med. Cell Longev..

[187] Nandini H.S., Krishna K.L., Apattira C. (2022). Combination of *Ocimum sanctum* extract and Levetiracetam ameliorates cognitive dysfunction and hippocampal architecture in rat model of Alzheimer’s disease. J. Chem. Neuroanat..

[188] Peng Y., Tao H., Wang S., Xiao J., Wang Y., Su H. (2021). Dietary intervention with edible medicinal plants and derived products for prevention of Alzheimer’s disease: A compendium of time-tested strategy. J. Funct. Foods.

[189] Hening P., Mataram Auriva M.B., Wijayanti N., Kusindarta D.L., Wihadmadyatami H. (2018). The neuroprotective effect of *Ocimum sanctum* Linn. ethanolic extract on human embryonic kidney-293 cells as In Vitro model of neurodegenerative disease. Vet. World.

[190] Oyem J.C., Chris-Ozoko L.E., Enaohwo M.T., Otabor F.O., Okudayo V.A., Udi O.A. (2021). Antioxidative properties of *Ocimum gratissimum* alters Lead acetate induced oxidative damage in lymphoid tissues and hematological parameters of adult Wistar rats. Toxicol. Rep..

[191] Udi O.A., Oyem J.C., Ebeye O.A., Chris-Ozoko L.E., Igbigbi P.S., Olannye D.U. (2022). The effects of aqueous extract of ocimum gratissimum on the cerebellum of male wistar rats challenged by lead acetate. Clin. Nutr. Open Sci..

[192] Berić T., Nikolić B., Stanojević J., Vuković-Gačić B., Knežević-Vukčević J. (2008). Protective effect of basil (*Ocimum basilicum* L.) against oxidative DNA damage and mutagenesis. Food Chem. Toxicol..

[193] Chenni M., El Abed D., Rakotomanomana N., Fernandez X., Chemat F. (2016). Comparative Study of Essential Oils Extracted from Egyptian Basil Leaves (*Ocimum basilicum* L.) Using Hydro-Distillation and Solvent-Free Microwave Extraction. Molecules.

[194] Farouk A., Fikry R., Saad M. (2016). Chemical Composition and Antioxidant Activity of *Ocimum basilicum* L. Essential Oil Cultivated in Madinah Monawara, Saudi Arabia and its Comparison to the Egyptian Chemotype. J. Essent. Oil Bear. Plants.

[195] Kaurinovic B., Popovic M., Vlaisavljevic S., Trivic S. (2011). Antioxidant Capacity of *Ocimum basilicum* L. and *Origanum vulgare* L. Extracts. Molecules.

[196] Kwee E.M., Niemeyer E.D. (2011). Variations in phenolic composition and antioxidant properties among 15 basil (*Ocimum basilicum* L.) cultivars. Food Chem..

[197] Nguyen P.M., Niemeyer E.D. (2008). Effects of Nitrogen Fertilization on the Phenolic Composition and Antioxidant Properties of Basil (*Ocimum basilicum* L.). J. Agric. Food Chem..

[198] Patil D., Mhaske D., Wadhawa G. (2011). Antibacterial and Antioxidant study of *Ocimum basilicum* Labiatae (sweet basil). J. Adv. Pharma. Ed. Res..

[199] Rameshrad M., Salehian R., Fathiazad F., Hamedeyazdan S., Garjani M., Maleki-Dizaji N., Vosooghi R. (2015). The effects of *Ocimum basilicum* ethanol extract on carrageenan induced paw inflammation in rats. Pharm. Sci..

[200] Bora K.S., Arora S., Shri R. (2011). Role of *Ocimum basilicum* L. in prevention of ischemia and reperfusion-induced cerebral damage, and motor dysfunctions in mice brain. J. Ethnopharmacol..

[201] Koutroumanidou E., Kimbaris A., Kortsaris A., Bezirtzoglou E., Polissiou M., Charalabopoulos K., Pagonopoulou O. (2013). Increased seizure latency and decreased severity of pentylenetetrazol-induced seizures in mice after essential oil administration. Epilepsy Res. Treat..

[202] Azmi N. (2018). A Brief Review of Potential Neuroprotective Roles of the Culinary Herb *Ocimum basilicum*. Med. Health.

[203] Shakeri F., Hosseini M., Ghorbani A. (2019). Neuropharmacological effects of *Ocimum basilicum* and its constituents. Physiol. Pharmacol..

[204] Singh V., Kahol A., Singh I.P., Saraf I., Shri R. (2016). Evaluation of anti-amnesic effect of extracts of selected Ocimum species using in-vitro and in-vivo models. J. Ethnopharmacol..

[205] Jamshidi N., Cohen M.M. (2017). The Clinical Efficacy and Safety of Tulsi in Humans: A Systematic Review of the Literature. Evid. Based Complement. Alternat Med..

[206] Sampath S., Mahapatra S.C., Padhi M.M., Sharma R., Talwar A. (2015). Holy basil (*Ocimum sanctum* Linn.) leaf extract enhances specific cognitive parameters in healthy adult volunteers: A placebo controlled study. Indian. J. Physiol. Pharmacol..

[207] Saxena R.C., Singh R., Kumar P., Negi M.P., Saxena V.S., Geetharani P., Allan J.J., Venkateshwarlu K. (2012). Efficacy of an Extract of *Ocimum tenuiflorum* (OciBest) in the Management of General Stress: A Double-Blind, Placebo-Controlled Study. Evid. Based Complement. Alternat Med..

[208] Bhattacharyya D., Sur T.K., Jana U., Debnath P.K. (2008). Controlled programmed trial of *Ocimum sanctum* leaf on generalized anxiety disorders. Nepal. Med. Coll. J..

[209] Lopresti A.L., Smith S.J., Metse A.P., Drummond P.D. (2022). A randomized, double-blind, placebo-controlled trial investigating the effects of an *Ocimum tenuiflorum* (Holy Basil) extract (Holixer^TM^) on stress, mood, and sleep in adults experiencing stress. Front. Nutr..

[210] Johansson L., Guo X., Waern M., Ostling S., Gustafson D., Bengtsson C., Skoog I. (2010). Midlife psychological stress and risk of dementia: A 35-year longitudinal population study. Brain.

[211] Sabia S., Fayosse A., Dumurgier J., van Hees V.T., Paquet C., Sommerlad A., Kivimäki M., Dugravot A., Singh-Manoux A. (2021). Association of sleep duration in middle and old age with incidence of dementia. Nat. Commun..

[212] Tadros M.G., Ezzat S.M., Salama M.M., Farag M.A. (2014). In vitro and in vivo Anticholinesterase Activity of the Volatile Oil of the Aerial Parts of *Ocimum basilicum* L. and O. africanum Lour. Growing in Egypt. Int. J. Pharmacol. Pharm. Sci..

[213] Wirtu S.F., Mishra A.K., Jule L.T., Ramaswamy K. (2024). *Ocimum basilicum* and *Ocimum americanum*: A Systematic Literature Review on Chemical Compositions and Antimicrobial Properties. Nat. Prod. Commun..

[214] Singh V., Krishan P., Singh N., Kumar A., Shri R. (2017). Amelioration of ischemia-reperfusion induced functional and biochemical deficit in mice by *Ocimum kilimandscharicum* leaf extract. Biomed. Pharmacother..

[215] Ayuob N.N., Balgoon M.J., Ali S., Alnoury I.S., ALmohaimeed H.M., AbdElfattah A.A. (2020). *Ocimum basilicum* (Basil) Modulates Apoptosis and Neurogenesis in Olfactory Pulp of Mice Exposed to Chronic Unpredictable Mild Stress. Front. Psychiatry.

[216] Singh V., Krishan P., Shri R. (2018). Improvement of memory and neurological deficit with *Ocimum basilicum* L. extract after ischemia reperfusion induced cerebral injury in mice. Metab. Brain Dis..

[217] Hao P.M., Quoc L.P.T. (2024). Chemical profile and antimicrobial activity of *Ocimum gratissimum* L. essential oil from Dak Lak province, Vietnam. J. Plant Biotechnol..

[218] Taguchi R., Hatayama K., Takahashi T., Hayashi T., Sato Y., Sato D., Ohta K., Nakano H., Seki C., Endo Y. (2017). Structure–activity relations of rosmarinic acid derivatives for the amyloid β aggregation inhibition and antioxidant properties. Eur. J. Med. Chem..

[219] Hole K.L., Williams R.J. (2021). Flavonoids as an Intervention for Alzheimer’s Disease: Progress and Hurdles Towards Defining a Mechanism of Action. Brain Plast..

[220] Liu J.Y., Guo H.Y., Quan Z.S., Shen Q.K., Cui H., Li X. (2023). Research progress of natural products and their derivatives against Alzheimer’s disease. J. Enzyme Inhib. Med. Chem..

[221] Chowdhury S., Kumar S. (2021). Inhibition of BACE1, MAO-B, cholinesterase enzymes, and anti-amyloidogenic potential of selected natural phytoconstituents: Multi-target-directed ligand approach. J. Food Biochem..

[222] Shibuya Y., Tsuzawa K., Onimaru H., Izumizaki M. (2024). Effects of linalool on respiratory neuron activity in the brainstem-spinal cord preparation from newborn rats. Biomed. Res..

[223] Kashyap D., Sharma A., Tuli H.S., Punia S., Sharma A.K. (2016). Ursolic Acid and Oleanolic Acid: Pentacyclic Terpenoids with Promising Anti-Inflammatory Activities. Recent. Pat. Inflamm. Allergy Drug Discov..

